# Two apples a day lower serum cholesterol and improve cardiometabolic biomarkers in mildly hypercholesterolemic adults: a randomized, controlled, crossover trial

**DOI:** 10.1093/ajcn/nqz282

**Published:** 2019-12-16

**Authors:** Athanasios Koutsos, Samantha Riccadonna, Maria M Ulaszewska, Pietro Franceschi, Kajetan Trošt, Amanda Galvin, Tanya Braune, Francesca Fava, Daniele Perenzoni, Fulvio Mattivi, Kieran M Tuohy, Julie A Lovegrove

**Affiliations:** 1 Hugh Sinclair Unit of Human Nutrition and the Institute for Cardiovascular and Metabolic Research, Department of Food and Nutritional Sciences, University of Reading, Reading, United Kingdom; 2 Department of Food Quality and Nutrition, Research and Innovation Centre, Fondazione Edmund Mach, San Michele all'Adige, Italy; 3 Unit of Computational Biology, Research and Innovation Centre, Fondazione Edmund Mach, San Michele all'Adige, Italy; 4 Steno Diabetes Centre Copenhagen, Gentofte, Denmark; 5 Department of Physics, University of Trento, Povo, Italy

**Keywords:** apple, polyphenols, flavanols, proanthocyanidins, fiber, lipid, cholesterol, vascular, bile acids, sex

## Abstract

**BACKGROUND:**

Apples are rich in bioactive polyphenols and fiber. Evidence suggests that consumption of apples or their bioactive components is associated with beneficial effects on lipid metabolism and other markers of cardiovascular disease (CVD). However, adequately powered randomized controlled trials are necessary to confirm these data and explore the mechanisms.

**OBJECTIVE:**

We aimed to determine the effects of apple consumption on circulating lipids, vascular function, and other CVD risk markers.

**METHODS:**

The trial was a randomized, controlled, crossover, intervention study. Healthy mildly hypercholesterolemic volunteers (23 women, 17 men), with a mean ± SD BMI 25.3 ± 3.7 kg/m^2^ and age 51 ± 11 y, consumed 2 apples/d [Renetta Canada, rich in proanthocyanidins (PAs)] or a sugar- and energy-matched apple control beverage (CB) for 8 wk each, separated by a 4-wk washout period. Fasted blood was collected before and after each treatment. Serum lipids, glucose, insulin, bile acids, and endothelial and inflammation biomarkers were measured, in addition to microvascular reactivity, using laser Doppler imaging with iontophoresis, and arterial stiffness, using pulse wave analysis.

**RESULTS:**

Whole apple (WA) consumption decreased serum total (WA: 5.89 mmol/L; CB: 6.11 mmol/L; *P* = 0.006) and LDL cholesterol (WA: 3.72 mmol/L; CB: 3.86 mmol/L; *P* = 0.031), triacylglycerol (WA: 1.17 mmol/L; CB: 1.30 mmol/L; *P* = 0.021), and intercellular cell adhesion molecule-1 (WA: 153.9 ng/mL; CB: 159.4 ng/mL; *P* = 0.028), and increased serum uric acid (WA: 341.4 μmol/L; CB: 330 μmol/L; *P* = 0.020) compared with the CB. The response to endothelium-dependent microvascular vasodilation was greater after the apples [WA: 853 perfusion units (PU), CB: 760 PU; *P* = 0.037] than after the CB. Apples had no effect on blood pressure or other CVD markers.

**Conclusions:**

These data support beneficial hypocholesterolemic and vascular effects of the daily consumption of PA-rich apples by mildly hypercholesterolemic individuals.

This trial was registered at clinicaltrials.gov as NCT01988389.

## Introduction

Regular consumption of plant foods, including fruits, has been consistently associated with a lower risk of cardiovascular disease (CVD) (1). Apples represent 12.5% of all consumed fruits in the world (2), due to their taste and convenience, and possibly also because of their purported health benefits. Epidemiological studies suggest that a frequent apple intake is inversely associated with acute coronary syndrome, total CVD mortality, and all-cause mortality (3, 4).

Apples are an excellent source of polyphenols (typically 110 mg/100 g) and fiber (typically 2–3 g/100 g) and these bioactive components may be responsible for the potential health effects (5). Apple polyphenols include flavanols [catechin and proanthocyanidins (PAs)] as the major class (71–90%), followed by hydroxycinnamates (4–18%), flavonols (1–11%), dihydrochalcones (2–6%), and anthocyanins (1–3%) which are found only in red apples (5). PAs and flavanol monomers, as extracts or within a food, have been shown to lower serum cholesterol, raise HDL cholesterol, inhibit LDL oxidation, activate endothelial nitric oxide synthase, prevent platelet aggregation, and block inflammatory responses in atherosclerosis, although data from human intervention studies are limited and sometimes contradictory (6–8). In addition, pectin, the main soluble fiber found in apples, is reported to affect transit time, gastric emptying, and nutrient absorption, affecting lipid and glucose metabolism (5). Pectin also appears to modulate the gut microbiota, a key determinant of bile acid (BA) chemical structure and thus signaling potential (5).

There is consistent evidence that apple consumption is associated with hypolipidemic effects, with reductions in total cholesterol (TC) and LDL cholesterol in animal models, but these studies cannot be directly extrapolated to humans (5). Human intervention studies also suggest potential lipid-lowering effects of apples but results lack consistency, perhaps due to differences in the apple products tested, which have included fresh whole apples (WAs) (9–16), dry apples (17, 18), apple skin (19), apple pomace (15), cloudy (15, 20, 21) and clear apple juice (11,15), and pure apple polyphenols (8, 22, 23); dose and molecular characteristics of the active components; study duration; sample size; and the use of appropriate controls (5). The effects of apples on endothelial function have been observed in postprandial human studies (19, 24) but are inconsistent in longer-term interventions (4-wk treatment) (17, 19), whereas effects on systemic inflammation are limited (5).

In this 8-wk dietary intervention study, we examined the hypothesis that the supplementation of the habitual diet of 40 healthy mildly hypercholesterolemic subjects with 2 fresh WAs (Renetta Canada) daily for 8 wk would result in TC reduction and beneficial effects on vascular function and other markers of CVD, compared with a sugar-matched apple control beverage (CB). Renetta Canada is an apple variety rich in PAs and dihydrochalcones and was previously shown to possess beneficial effects on gut microbiota composition and activity in vitro (25).

## Methods

### Research ethics

The study (NCT01988389) was given a favorable ethical opinion for conduct by the University of Reading Research Ethics Committee [UREC Project No. 13/22 (AVAG/effect of Apples on cardioVascular risk And Gut health study)]. The study was conducted according to the guidelines laid down in the Declaration of Helsinki of 1975 as revised in 1983. Subjects provided written informed consent before participating and a small remuneration was given for participation in the trial.

### Participants

**FIGURE 1:**
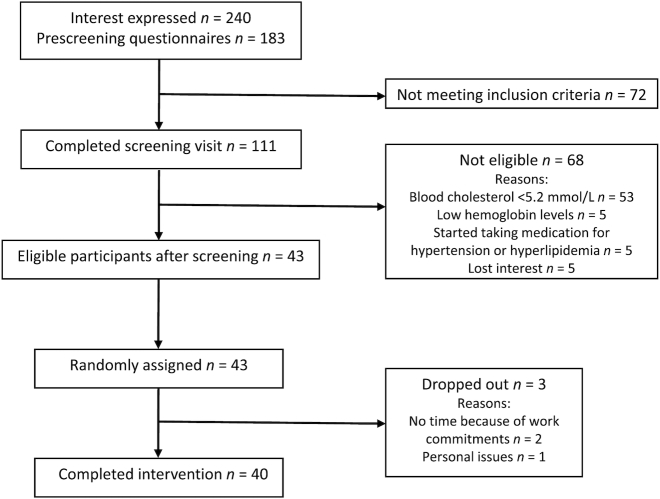
Participant flow diagram of the AVAG (effect of Apples on cardioVascular risk And Gut health) study.

Forty-three generally healthy volunteers (18 men, 25 women), aged 29–69 y, with BMI (in kg/m^2^) 19–33 and TC > 5.2 mmol/L (mildly hypercholesterolemic), were recruited from the general population of Reading, Berkshire, United Kingdom using human volunteer databases, posters, and adverts on social media (**Figure 1**). Subjects first completed a health and lifestyle questionnaire and then attended the Hugh Sinclair Unit of Human Nutrition at the University of Reading in a fasted state. After giving informed written consent, screening measurements were performed, such as height, weight, blood pressure, and blood measurements including lipid profile, liver and kidney function markers, and full blood count, 2–4 wk before commencing the trial. Subjects with an abnormal blood biochemistry profile, based on standard clinical cutoffs, were excluded. Further exclusion criteria included medical history of heart disease (or family history) and diabetes mellitus; kidney, liver, or pancreatic diseases; hematological and gastrointestinal disorders; and medication for hyperlipidemia, hypertension, hypercoagulation, inflammation, and gastrointestinal motility. Other exclusion criteria were pregnancy (current or planned) or lactation, smoking, history of alcohol misuse, antibiotic intake <3 mo before the study, food supplement use (including fish oil, vitamins, phytochemicals, probiotics, and prebiotics unless willing to stop 2 mo before study commencement), habitual intake of >1 apple/d, vegetarian and vegan diet, and planned weight loss regime.

The primary outcome was changes in serum cholesterol. All other outcomes were secondary and included the evaluation of endothelial function, measured by laser Doppler imaging (LDI), changes in blood pressure and arterial stiffness, measured with the use of an ambulatory blood pressure monitor and pulse wave analysis (PWA), respectively, and changes in serum lipids, BAs, markers of inflammation, endothelial function, glycemic control, and insulin resistance.

### Study design

**FIGURE 2:**
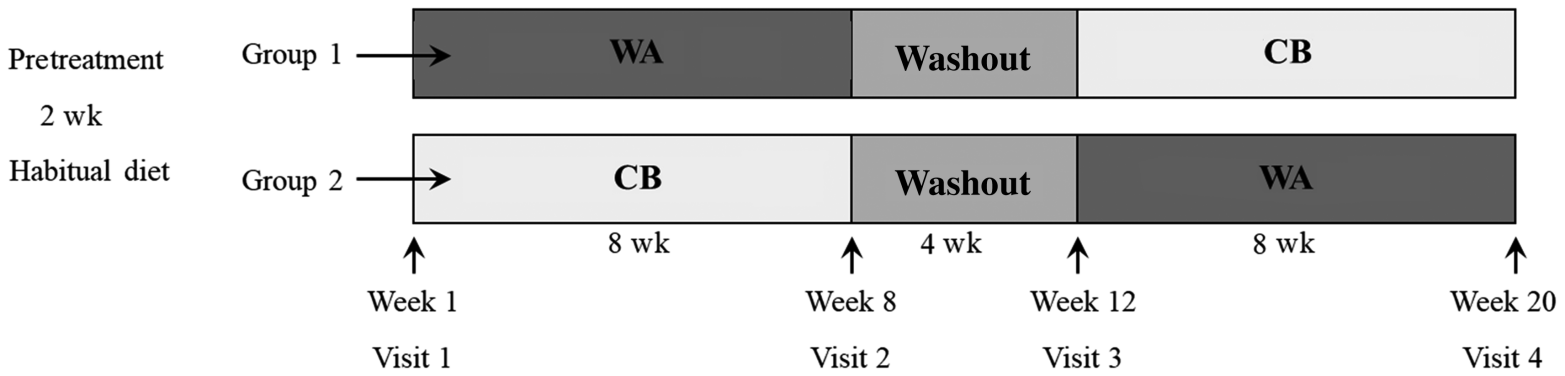
Study design of a randomized, controlled, crossover study in which 40 healthy volunteers (Group 1: *n* = 22; Group 2: *n* = 18) received daily 2 WAs (340 g without core) or a CB (100 mL concentrated + 400 mL water) for a period of 8 wk each, separated by a 4-wk washout period. Fasted blood and a 24-h urine sample were collected on each study visit (weeks 1, 8, 12, and 20), and ambulatory blood pressure, vascular reactivity, and arterial stiffness were assessed. CB, control beverage; WA, whole apple.

The present trial was a randomized, controlled, crossover, dietary intervention study performed between November 2013 and August 2014. Forty-three volunteers were recruited onto the study; 3 volunteers dropped out due to personal reasons and 40 subjects (17 men, 23 women) completed the trial (Figure 1). For a 2-wk run-in period before the dietary intervention, volunteers followed their habitual diet but were required not to consume probiotics (e.g., live yogurts, fermented milk drinks), prebiotics (e.g., inulin, fructooligosaccharide), and any apples, apple juice, or apple-containing foods. Probiotics, prebiotics, and any other apple products (apart from the provided items) were also strictly avoided throughout the study period (20 wk). Volunteers were randomly allocated into 2 groups, using a minimization program (minim) (26), stratified by age [23–45 y (*n* = 13), 46–69 y (*n* = 27)], sex (17 men, 23 women), BMI [<25 (*n* = 20), >25 (*n* = 20)], and TC [5.2–6.2 mmol/L (*n* = 26), >6.2 mmol/L (*n* = 14)], to receive daily either 2 WAs or a sugar-matched CB (**Table 1**, **Figure 2**). One group [*n* = 22 (10 men, 12 women); mean ± SD age: 51 ± 11 y; BMI: 25 ± 3; TC: 5.9 ± 0.7 mmol/L] consumed 2 apples/d (340 g without core) for 8 wk, and then after a 4-wk washout period, consumed 500 mL of a sugar-matched apple CB (100 mL concentrate + 400 mL water) daily for a further 8 wk, whereas the other group [*n* = 18 (7 men, 11 women); age: 52 ± 11 y; BMI: 26 ± 4; TC: 6.0 ± 0.6 mmol/L] received the intervention foods in the reverse order (Figure 2). Volunteers were asked to incorporate the intervention products into their normal habitual diet, and understood that both products had potential benefit. Clear instructions were given to eat the apple skin and flesh and not to consume the apple core.

**TABLE 1:** Nutritional composition of the intervention products (daily portion)^1^

Component	Whole apples^2^	Control beverage^3^
Energy,^4^ kJ	925	699
Energy,^4^ kcal	221	167
Fat,^4^ g	0.7	<0.1
Protein,^4^ g	1	<0.1
Carbohydrate total,^4^ g	58	42
Carbohydrate available,^4^ g	49	42
Total sugar,^4^ g	44	41
Glucose,^4^ g	8	18
Fructose,^4^ g	23	20
Sucrose,^4^ g	12	3
Total dietary fiber^4^ (AOAC), g	8.5	<0.5
Soluble fiber^4^ (AOAC), g	3.7	<0.5
Insoluble fiber^4^ (AOAC), g	4.8	<0.5
Vitamin C,^4^ mg	21	<3
Chlorogenic acid,^5^ mg	83 ± 8.9	0.9 ± 2.1
Cryptochlorogenic acid,^5^ mg	0.8 ± 0.4	0.1 ± 0.6
Ellagic acid,^5^ mg	0.2 ± 0.1	0 ± 0
Phloridzin,^5^ mg	13.8 ± 3.5	0.3 ± 0.6
Quercetin-3-*O*-arabinoside,^5^ mg	2.3 ± 1.0	0.03 ± 0.2
Quercetin-3-*O*-glucoside,^5^ mg	2.3 ± 1.9	0.03 ± 0.2
Quercetin-Glc-Rha (rutin),^5^ mg	1.2 ± 1.5	0.03 ± 0.2
Catechin,^5^ mg	5.3 ± 2.3	0.01 ± 0.03
Epicatechin,^5^ mg	27 ± 6.5	0.02 ± 0.09
Procyanidin B1,^5^ mg	23 ± 5.6	0.01 ± 0.03
Procyanidin B2,^5^ mg	176 ± 26	0.06 ± 0.32
Oligomeric PAs (DP > 2)^6^	854 ± 233	1 ± 1.7
Mean DP^6^	7.1	3.5
Sum of polyphenols^7^	990 ± 259	2.5 ± 5.7

1 Values are means ± SDs for polyphenols.

2 Consists of a 340-g daily serving (2 apples; Renetta Canada) without the core including skin.

3 Consists of a 100-mL control beverage + 400-mL water daily serving (sugar-matched control).

4 Measured by Campden BRI laboratories, UK, mixture of 3 apples.

5 Quantified in house according to Vrhovsek et al. (27), measurement of 15 apples from December to April.

6 Quantified in house according to Gris et al. (28), measurement of 15 apples from December to April. DP, degree of polymerization; PA, proanthocyanidin.

7 Sum of all individual polyphenols and oligomeric PAs (DP > 2).

The study included 4 main visits before and after each intervention period (weeks 1, 8, 12, and 20) (Figure 2). Twenty-four hours before each study visit, volunteers were asked to collect their urine and refrain from intensive exercise, alcohol, oily fish, and caffeine intake (e.g., coffee, tea). The night before they consumed a standardized low-fat, low-flavonoid meal followed by an overnight fast consuming only water. The volunteers were asked to attend the Hugh Sinclair Unit of Human Nutrition in the morning of each of the 4 study visit days in a fasted state. During these visits, several measurements were taken, including anthropometrics (height, weight, waist circumference), body fat composition (body composition analyzer, Tanita BC-418 digital scale), blood pressure (30-min measurement), arterial stiffness (PWA), and microvascular reactivity (LDI with iontophoresis). Moreover, volunteers returned their 24-h urine collection and a fasted blood sample was collected before they were offered breakfast. Volunteers received the intervention products on the visit day, and then every fortnight when they were also asked if there had been any changes to their lifestyle, such as diet, exercise levels, and medication. Finally, compliance was assessed with the daily completion of a tick sheet.

### Intervention products

Fresh apples from the same harvest (green variety “Renetta Canada”) grown in Val di Non, Trentino, Italy were used as the intervention food. All apples were kindly provided by Melinda SCA, Cles, Trentino. A total of 7337 apples were delivered to the University of Reading, United Kingdom in 7 batches between November 2013 and May 2014. There was no difference in the PA concentration of the apples during storage and between the different batches throughout the study (**Supplemental Figure 1**). After collection, the apples were stored in the dark at 4°C for ≤4–6 wk. The CB was a commercially available apple juice squash consisting of 50% apple juice from concentrate and added sugar which matched the total sugar content of the apples. The daily dietary content of the intervention products is shown in Table 1. The apples’ nutritional composition was analyzed by Campden BRI laboratories, United Kingdom and the detailed polyphenol content was measured in the laboratory of Fondazione Edmund Mach according to the methods of Gris et al. (28) and Vrhovsek et al. (27).

### Diet diary analysis

Participants were asked to complete a detailed 4-d diary of all foods and drinks consumed (3 weekdays and 1 day at the weekend), the week before each of the 4 main visits. The food diaries were analyzed using Dietplan6 (Forestfield Software Ltd.) to determine the macro- and micronutrient content of the participants’ diet based on UK food databases (29). Flavonoid intake was assessed by using the USDA Flavonoid Database and by an “in-house” compositional analysis of fruits and vegetables as described by Chong et al. (30).

### Anthropometric, body fat composition, and blood pressure analysis

Height, weight, waist circumference, body fat percentage, and blood pressure were measured on 4 occasions, at the beginning and the end of the 2 intervention periods. Body fat composition (percentage) was measured with a Tanita body composition monitor (Tanita BC-418 digital scale, Tanita Europe) based on bioelectrical impedance technology. Measurements were performed under normal settings with participants wearing light clothing. Blood pressure was recorded using an ambulatory blood pressure monitor which was adjusted to take a measurement every 5 min. Participants were relaxed, seated in a quiet, light room for 15 min before the commencement of the measurement, which lasted for 30 min and included 6 measurements.

### Assessment of vascular function

All measurements were performed in a quiet temperature-controlled room (mean ± range: 22 ± 1°C), with participants in a supine position after a 30-min rest. Microvascular reactivity was measured using a moorLDI2 laser Doppler Imager with iontophoresis (Moor Instruments Ltd.) as previously described (31). Briefly, acetylcholine chloride (2.5 mL, 1% in 0.5% NaCl solution) for the measurement of endothelium-dependent reactivity and sodium nitroprusside (2.5 mL, 1% in 0.5% NaCl solution) for the measurement of endothelium-independent reactivity were delivered transdermally on the forearm via iontophoresis using ION6 chambers (Moor Instruments). Current delivery was controlled and repeated scans were taken, giving a total charge of 8 millicoulombs. Microvascular response was calculated by using the area under the flux-versus-time curve (AUC), measured as perfusion units (arbitrary). PWA was measured using a high-fidelity applanation tonometer to detect and capture electronically the shape of the radial arterial pulse (SphygmoCor system, AtCor Medical). The radial artery pressure waveform was then calibrated with the brachial systolic and diastolic pressure measurements (performed in the traditional manner at the brachial artery with an inflated cuff). The SphygmoCor system then converted this peripheral waveform to a central aortic pressure waveform using general transfer function algorithms (32). Moreover, several indexes were quantified from the peripheral and central waveforms: central systolic pressure, central diastolic pressure, central pulse pressure, central mean pressure, heart rate (HR), and central augmentation pressure-to-pulse height ratio (C-AGPH)—also known as the Augmentation Index (AIx) or arterial stiffness. Given that the AIx depends on HR, it was corrected for an HR of 75 beats per minute (C-AGPH-HR75). Measurements were performed in triplicate by a single trained operator.

### Blood and urine sample collection

For each volunteer, blood samples were collected into separate vacutainer tubes (Greiner Bio-One Ltd.), containing EDTA or lithium heparin (LH) anticoagulants for plasma and a clot activator for serum [serum separator tubes (SSTs)]. After collection, the EDTA and LH tubes were immediately put on ice for transportation to the laboratory. Cells were then removed from plasma by centrifugation at 1800 × *g* for 15 min at 4°C. Aliquots (0.5 mL) of the supernatant (plasma) were immediately transferred into clean cryogenic (polypropylene) vials and stored at −80°C. To obtain serum, SSTs were left undisturbed at room temperature for 30 min after collection. The supernatant (serum) was then obtained by centrifugation at 1800 × *g* for 15 min at 21°C and aliquots were put into separate cryogenic vials for storage at −20°C.

For the collection of the urine, volunteers were provided with a urine pot containing 10 mL 1 M hydrochloric acid, ice packs, and an isothermic bag and they were instructed to start collecting the sample the morning before the study visit and for 24 h. Once the urine was received in the lab, its pH was reduced to 4 with the further addition of 1 M hydrochloric acid, then it was centrifuged at 1800 × *g* for 10 min at 4°C, divided into aliquots, and stored at −80°C until further analysis. Urine volume was also recorded. Analysis of blood and urine was performed when the intervention study was completed. All samples from each subject were analyzed together to reduce interbatch variation.

### Biochemical analysis

Concentrations of serum lipids [total cholesterol (TC), HDL cholesterol, triacylglycerol (TAG), and nonesterified fatty acids (NEFAs)], glucose, albumin, and uric acid were measured using an Automatic Analyzer ILAB 600 with appropriate enzyme-based kits (Instrumentation Laboratory Ltd.) and quality controls (Alpha Laboratories Ltd.). Controls were run with each batch and their values were within the range specified by the manufacturers. LDL cholesterol was estimated by using the Friedewald formula (LDL cholesterol = TC – HDL cholesterol − TAG/2.19). ELISA kits were used for the determination of serum insulin (Dako Ltd.), adiponectin, and endothelin (R&D Systems Europe Ltd.) according to the manufacturer's instructions. An Adhesion Molecule Luminex Performance Assay 4-plex kit was used for the determination of E-selectin, P-selectin, vascular cell adhesion molecule-1 (VCAM-1), and intercellular cell adhesion molecule-1 (ICAM-1) (R&D Systems Europe Ltd.) using a flow cytometry–based Luminex 200^TM^ System (Invitrogen, Thermo Fisher Scientific). A Milliplex MAP human cytokine kit (Merck-Millipore) was used for the determination of TNF-α, IL-1β, and IL-6 according to the manufacturer's instructions. IL-1β and IL-6 were under the detection limit and are not reported here.

### BAs in blood

BAs were quantified by the isotopic dilution method described elsewhere (33) and modified for the purpose of the current sample set. Briefly, 100 μL plasma was placed in Sirocco 96-well plates followed by 200 μL internal standards in methanol and 200 μL methanol:water (90:10, vol:vol) with 0.1% formic acid. Samples were filtered and the residue was extracted with 400 μL acetonitrile:acetone (80:20, vol:vol) with 0.1% formic acid. The extract was evaporated until dryness and resolved with 200 μL methanol:water (50:50, vol:vol) before injection. Samples were analyzed using a UHPLC (ultra-HPLC) UltiMate 3000 (Dionex) coupled with a Triple Quad 5500 (AB Sciex) mass spectrometer. Data were acquired according to multiple reaction monitoring methodology and results were expressed using an appropriate internal standard. Raw data were processed by AB Sciex Analyst 1.6.1 software. A detailed method description and validation can be found in the **Supplemental Methods**. The ursodeoxycholic acid was excluded from the statistical analysis because >30% of measures were missing. Missing values of glycolithocholic acid (20 measures, distributed among 8 subjects) and glycoursodeoxycholic acid (GUDCA) (4 measures, distributed among 2 subjects) were imputed with a random number ranging between 0 and the limit of quantification for each compound (Supplemental Methods, **Supplemental Tables 1–3**).

### Phloretin metabolites in urine

Urine samples were submitted to untargeted chemical analysis for the identification of phloretin metabolites as biomarkers of apple consumption (Supplemental Methods) (34). Phloretin glucuronide was not detected in 16% of measures and phloretin glucuronide sulfate in 61% of measures; these were detected almost solely in the presence of apple intake. Missing values were imputed with a random number ranging between 0 and 5,000, which is compatible with the electrical noise of the mass spectrometer.

### Statistics

The primary outcome of the study was to measure relative changes in serum TC and it was calculated that 38 volunteers were required to detect a 5% change (*P* < 0.05 and 90% power), assuming a 6-mmol/L mean TC with a 0.41-mmol/L within-subject SD. On the basis of this power calculation, 43 volunteers were recruited to allow for a 10% dropout rate. Samples from each participant were recoded to protect volunteer identity and to mask treatment groups during the analysis.

Exploratory analysis on the study primary outcome (TC) was initially performed by calculating, for each individual, the difference (Δ*C*) in TC after the consumption of WAs and CB. Intervention-specific baselines were also taken into account [Δ*C* = (*C*_apple_ − *C*_apple baseline_) − (*C*_control_ − *C*_control baseline_)]. The presence of a significant difference in the TC concentration was then tested by a 1-sample *t* test against a null hypothesis of Δ*C* = 0. The dependence of Δ*C* on the main study factors (age, BMI, and sex) was also assessed in this phase (**Supplemental Figures 2–4**).

Differences between the dietary components of apples and the CB were assessed using a *t* test, whereas the PA content of apples throughout storage was assessed using an ANOVA test using IBM SPSS version 21 (SPSS Inc.).

In order to test the difference in treatment response means, baseline and postbaseline data were jointly modeled applying mixed-effect linear modeling, accounting for subject variability (35). The model assumes that the effects of systematic bias typical of crossover studies, such as carryover, are negligible. As suggested for the 2-period 2-treatment design (35), a formal test on carryover was not performed. Restricted maximum likelihood was used to estimate the model parameters. To perform model optimization, the distribution of the conditional residuals of the models was inspected to assess normality and homoscedasticity, and exclude the presence of groups. To improve the distribution of skewed variables and the conditional residuals of the model, log_10_ transformation was applied. For these variables, the model estimates for both baselines and treatments are presented as back-transformed values, which correspond to the median measured concentrations, in order to facilitate the interpretation of the results and allow their direct comparison with published clinical ranges. The uncertainty on the inference estimates was calculated with a bootstrap operation (500 repeats) and is indicated as (possibly back-transformed) 95% CIs.

In order to provide all the information to properly interpret the study findings, the treatment effect (computed as the difference between the value of a variable after WA intake and after CB consumption), as well as the estimated sequence and period effect, and the number of observations and subjects used for building each model are also explicitly reported (**Supplemental Tables 4–7**) (36). These results were not back-transformed from the logarithmic scale in order to improve interpretation. It is worth noting that with our modeling strategy the significance of the treatment effect can also be reliably assessed in the presence of period/sequence effects, even if the estimation of the effect is suboptimal (37).

The mixed linear models were built in R (www.r-project.org) version 3.5.0 by using packages *lme4* version 1.1-17 (38) and *lmerTest* version 3.0-1 (39), whereas the CIs were computed using the package *stats. P* values were computed using the Kenward-Roger approximation and 0.05 was chosen as the significance threshold.

The association between TC and BAs in women was tested without taking into account baselines. The predictors were chosen from among the BAs, which were log transformed, centered, and scaled using the R package *LaplacesDemon* version 16.1.0. The study design factors (age, treatment, sequence, period) were also included in the model and age was centered and scaled in the same way as the BAs. Both *R*^2^ and Ω^2^ indicate that the model fit is good, being 0.961 and 0.960, respectively. The use of a mixed linear model also allowed us to take into account subject variability together with the grouping of the samples within the 2 sequences of the crossover design. In particular, subject nesting within the sequence was defined as a random effect.

## Results

### Nutritional composition analysis of intervention products

Table 1 presents the detailed nutritional composition analysis of the intervention products. Apples contained higher amounts of polyphenols and fiber than the CB (*P* < 0.05). In particular, a daily dose of 340 g apples provided 990 mg polyphenols (presented as the sum of the individual phenolic compounds), 854 mg PAs, and 8.5 g total fiber (3.7 g soluble) compared with 2.5 mg polyphenols, 1 mg PAs, and <0.5 g total fiber from the daily intake of 500 mL sugar-matched CB (Table 1).

### Study population characteristics, dietary intakes, and compliance

**FIGURE 3:**
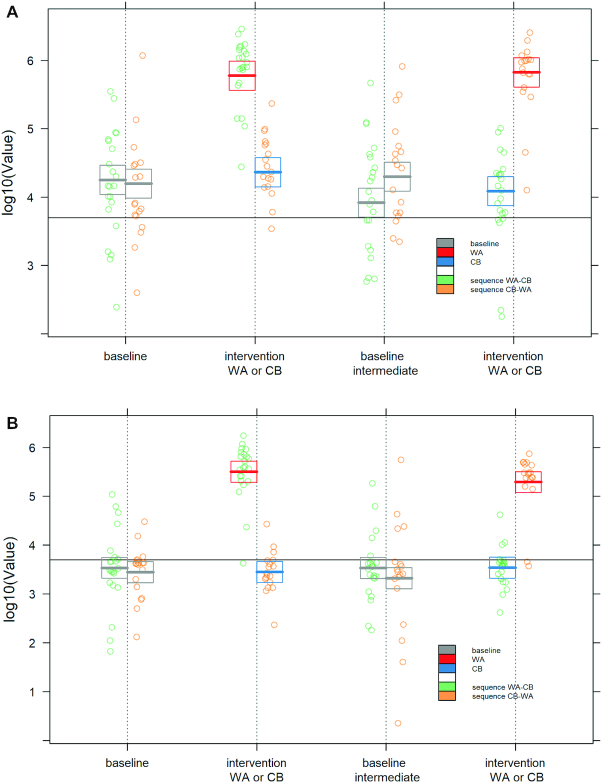
Measured values of urinary phloretin glucuronide (A) and phloretin glucuronide sulfate (B) for each visit, for both the treatments. Dots are individual values. Vertical dotted lines intersecting the *x* axis represent each visit; sequence WA–CB is plotted to the left of the vertical dotted lines and sequence CB–WA to the right of them. The boxplots represent the mean values and the 95% bootstrap CIs as computed by the mixed linear models of treatment effect. Red: estimates corresponding to WA intake; blue: estimates corresponding to CB intake; gray: estimates corresponding to both baselines (initial and intermediate). CB, control beverage; WA, whole apple.

Of the 43 volunteers who started the study, 40 completed all visits (23 women, 17 men). Three participants dropped out for personal reasons. No adverse effects were reported during the study. The volunteers’ age range was 29–69 y (mean: 51 y) and BMI was between 18.4 and 33.4 kg/m^2^ (mean: 25.3). Volunteers had mean TC above that considered physiologically normal (6.08 mmol/L compared with guideline values of <5.2 mmol/L). **Table 2** and Supplemental Table 4 show the baseline measurements before the 2 treatments. There was no effect of the intervention on body weight, BMI, waist circumference, and body fat percentage throughout the 20-wk period (Table 2, Supplemental Table 4). There was no change in daily energy and dietary carbohydrates, sugars, fat, and protein (**Table 3**, Supplemental Table 5). During the WA intervention, daily dietary fiber (*P* = 0.001) and flavonoid intake (*P* = 0.001) increased compared with the CB, suggesting that the participants complied with the dietary protocol (Table 3, Supplemental Table 5). Compliance was further confirmed by the higher concentrations of urinary biomarkers of apple intake in the WA group compared with the CB group (*P* < 0.05). **Figure 3** shows the log10 value of intensities for urinary phloretin glucuronide (Figure 3A) and phloretin glucuronide sulfate (Figure 3B) measured in urine samples throughout the intervention. Phloretin glucuronide and phloretin glucuronide sulfate were detected almost exclusively after WA intake.

**TABLE 2:** Anthropometric characteristics and blood biochemistry in mildly hypercholesterolemic participants BT (week 1) and AT (week 8) with WAs or CB^1^

			WAs		CB		
Parameters	L	*n*/*N*	BT (95% CI)	AT (95% CI)	BT (95% CI)	AT (95% CI)	*P* value
Anthropometrics							
Weight, kg		160/40	72.1 (67.66, 76.58)	72.3 (67.86, 76.78)	72.2 (67.74, 76.66)	72.4 (67.96, 76.88)	0.677
BMI, kg/m^2^		160/40	25.3 (24.15, 26.51)	25.4 (24.22, 26.57)	25.3 (24.17, 26.53)	25.4 (24.27, 26.63)	0.500
Waist, cm		144/39	88.5 (85.01, 92.05)	88.5 (85.01, 92.05)	89.0 (85.51, 92.53)	88.5 (84.98, 92.00)	0.895
Fat, %		152/40	28.8 (26.49, 31.18)	29.2 (26.87, 31.55)	29.0 (26.68, 31.36)	29.0 (26.67, 31.35)	0.484
Trunk fat, %		150/40	28.1 (26.01, 30.16)	28.6 (26.51, 30.67)	28.2 (26.09, 30.24)	28.2 (26.12, 30.27)	0.300
Blood biochemistry							
Total cholesterol, mmol/L		152/38	6.08 (5.84, 6.33)	5.89 (5.64, 6.14)	6.05 (5.80, 6.30)	6.11 (5.86, 6.36)	0.006
LDL cholesterol, mmol/L		152/38	3.89 (3.67, 4.11)	3.72 (3.50, 3.95)	3.83 (3.61, 4.05)	3.86 (3.64, 4.08)	0.031
HDL cholesterol, mmol/L		152/38	1.62 (1.49, 1.75)	1.58 (1.46, 1.71)	1.62 (1.50, 1.75)	1.61 (1.48, 1.74)	0.246
TAG, mmol/L	✓	152/38	1.17 (1.04, 1.32)	1.17 (1.04, 1.32)	1.19 (1.06, 1.35)	1.30 (1.15, 1.46)	0.021
NEFAs, μmol/L	✓	152/38	456.9 (402.23, 518.98)	431.3 (379.74, 489.97)	456.3 (401.72, 518.32)	426.1 (375.09, 483.96)	0.807
Glucose, mmol/L		152/38	5.03 (4.88, 5.17)	5.04 (4.89, 5.18)	5.14 (5.00, 5.29)	5.09 (4.95, 5.24)	0.229
Insulin, pmol/L	✓	160/40	33.8 (28.80, 39.79)	34.3 (29.15, 40.27)	35.9 (30.51, 42.16)	38.7 (32.89, 45.45)	0.051
TNF-α, pg/mL		160/40	6.5 (5.69, 7.31)	6.1 (5.30, 6.92)	6.2 (5.44, 7.06)	6.5 (5.70, 7.33)	0.106
Albumin, g/L		152/38	44.1 (43.32, 44.94)	43.3 (42.51, 44.13)	44.2 (43.43, 45.06)	43.8 (42.95, 44.57)	0.177
Adiponectin, μg/mL	✓	152/38	6.4 (5.10, 8.11)	6.6 (5.20, 8.26)	6.4 (5.04, 8.01)	6.2 (4.92, 7.81)	0.071
Endothelin, pg/mL	✓	160/40	1.1 (1.04, 1.26)	1.2 (1.06, 1.27)	1.1 (1.02, 1.23)	1.1 (1.03, 1.24)	0.561
Uric acid, μmol/L		152/38	337.3 (313.65, 360.88)	341.4 (317.75, 364.98)	338.1 (314.46, 361.69)	330.0 (306.41, 353.64)	0.020
VCAM-1, ng/mL	✓	152/38	696.6 (638.88, 759.55)	665.7 (610.56, 725.88)	682.6 (626.05, 744.30)	690.8 (633.60, 753.28)	0.090
ICAM-1, ng/mL	✓	152/38	152.6 (133.04, 174.94)	153.9 (134.22, 176.48)	156.5 (136.44, 179.41)	159.4 (139.04, 182.83)	0.028
E-selectin, ng/mL	✓	152/38	32.1 (28.82, 35.72)	32.8 (29.45, 36.50)	32.4 (29.11, 36.09)	33.3 (29.90, 37.07)	0.417
P-selectin, ng/mL	✓	152/38	34.7 (32.16, 37.34)	34.7 (32.17, 37.35)	35.6 (33.06, 38.39)	35.2 (32.69, 37.95)	0.214

1 Values are means (for nontransformed variables mean is equal to median) or medians (for the log-transformed variables) with (500) bootstrap 95% CIs estimated with a joint mixed-effect linear model on BT and AT data, adjusted for subject variability. The treatment effect is statistically significant when *P* < 0.05. AT, after treatment; BT, before treatment; CB, control beverage; ICAM-1, intercellular cell adhesion molecule-1; L, the model was built using the log10-transformed variable and the values are back-transformed in the original scale for the reader's convenience; *n*, number of observations; *N*, number of subjects; NEFA, nonesterified fatty acid; TAG, triacylglycerol; VCAM-1, vascular cell adhesion molecule-1; WA, whole apple.

**TABLE 3:** Dietary nutrient intake in mildly hypercholesterolemic participants BT (week 1) and AT (week 8) with WAs or CB^1^

			WAs		CB		
Parameters	T	*n*/*N*	BT (95% CI)	AT (95% CI)	BT (95% CI)	AT (95% CI)	*P* value
Energy, kJ	L	156/39	7396 (6716, 8144)	8195 (7442, 9024)	7363 (6687, 8108)	8097 (7353, 8917)	0.680
Energy, kcal	L	156/39	1769 (1606, 1949)	1947 (1768, 2145)	1749 (1588, 1927)	1926 (1749, 2122)	0.708
Carbohydrates, g		156/39	194 (174, 214)	241 (221, 261)	198 (178, 218)	229 (209, 249)	0.144
Total sugar, g		156/39	91.3 (79.8, 102.9)	119.6 (108.1, 131.2)	84.8 (73.3, 96.4)	122.2 (110.7, 133.8)	0.670
Fat, g	L	156/39	67.8 (59.9, 76.7)	69.5 (61.4, 78.6)	66.2 (58.5, 74.9)	69.5 (61.5, 78.7)	0.983
Protein, g	L	156/39	71.9 (65.8, 78.7)	75.4 (69.0, 82.5)	72.9 (66.6, 79.7)	74.1 (67.8, 81.0)	0.637
Fiber (AOAC), g		156/39	20.6 (18.3, 22.8)	26.9 (24.6, 29.2)	20.5 (18.2, 22.8)	20.3 (18.0, 22.5)	0.001
Total flavonoids, mg	S	156/39	438 (298, 603)	1534 (1263, 1833)	517 (365, 696)	426 (289, 589)	0.001

1 Values are means (for nontransformed variables mean is equal to median) or medians (for the log- or square root-transformed variables) with (500) bootstrap 95% CIs estimated with a joint mixed-effect linear model on BT and AT data, adjusted for subject variability. The treatment effect is statistically significant when *P* < 0.05. AT, after treatment; BT, before treatment; CB, control beverage; *n*, number of observations; *N*, number of subjects; T, the model was built using a transformed version of the variable (L = log10, S = square root) and the values are back-transformed in the original scale for the reader's convenience; WA, whole apple.

### Biochemical analysis

Table 2 and Supplemental Table 4 show the blood biochemical parameters. WA decreased serum TC (*P* = 0.006), LDL cholesterol (*P* = 0.031), and TAG (*P* = 0.021) compared with the CB. The WA intervention increased uric acid (*P* = 0.020) compared with the CB. There were no differences in the concentrations of circulating HDL cholesterol, NEFAs, glucose, insulin, TNF-α, albumin, adiponectin, and endothelin (Table 2, Supplemental Table 4). There was no effect of treatment on plasma BA concentrations (**Table 4**, Supplemental Table 6).

**TABLE 4:** Fasted circulating plasma bile acid concentrations in mildly hypercholesterolemic participants BT (week 1) and AT (week 8) with WAs or CB^1^

			WAs		CB		
Parameters	L	*n*/*N*	BT (95% CI)	AT (95% CI)	BT (95% CI)	AT (95% CI)	*P* value
Cholic acid, nM	✓	160/40	55.4 (36.9, 83.2)	41.5 (27.6, 62.3)	38.7 (25.8, 58.2)	49.0 (32.6, 73.6)	0.346
Chenodeoxycholic acid, nM	✓	160/40	44.4 (29.7, 66.3)	34.0 (22.8, 50.8)	35.2 (23.6, 52.5)	40.0 (26.8, 59.7)	0.344
Deoxycholic acid, nM	✓	160/40	93.9 (67.4, 130.7)	98.5 (70.7, 137.1)	99.1 (71.2, 137.9)	105.4 (75.7, 146.7)	0.603
Lithocholic acid, nM	✓	160/40	5.9 (5.0, 6.9)	6.1 (5.1, 7.1)	6.1 (5.2, 7.2)	6.4 (5.4, 7.5)	0.523
Glycocholic acid, nM	✓	160/40	86.8 (64.3, 117.2)	62.9 (46.6, 84.8)	73.0 (54.1, 98.5)	62.5 (46.3, 84.3)	0.967
Glycochenodeoxycholic acid, nM	✓	160/40	226.1 (164.5, 310.9)	191.1 (139.0, 262.7)	184.0 (133.9, 253.0)	188.6 (137.2, 259.2)	0.928
Glycodeoxycholic acid, nM	✓	160/40	79.8 (54.0, 117.8)	76.9 (52.1, 113.6)	70.2 (47.6, 103.7)	66.0 (44.7, 97.5)	0.261
Glycolithocholic acid, nM	✓	140/38	6.2 (4.7, 8.2)	5.1 (3.9, 6.7)	5.0 (3.8, 6.6)	4.9 (3.7, 6.4)	0.815
Glycoursodeoxycholic acid, nM	✓	156/40	23.2 (15.4, 35.0)	20.2 (13.4, 30.5)	21.0 (13.9, 31.6)	21.7 (14.4, 32.6)	0.656
Taurocholic acid, nM	✓	160/40	12.3 (9.0, 16.8)	9.4 (6.9, 12.8)	10.4 (7.6, 14.2)	9.3 (6.8, 12.7)	0.959
Taurochenodeoxycholic acid, nM	✓	160/40	25.8 (18.6, 35.6)	22.1 (16.0, 30.5)	23.8 (17.2, 32.9)	22.6 (16.4, 31.3)	0.847
Taurodeoxycholic.acid, nM	✓	160/40	13.8 (9.8, 19.4)	12.5 (8.9, 17.5)	12.2 (8.7, 17.2)	11.2 (8.0, 15.7)	0.424
Taurolithocholic acid, nM	✓	160/40	2.3 (2.1, 2.7)	2.3 (2.0, 2.6)	2.2 (2.0, 2.5)	2.1 (1.8, 2.3)	0.235

1 Values are means (for nontransformed variables mean is equal to median) or medians (for the log-transformed variables) with (500) bootstrap 95% CIs estimated with a joint mixed-effect linear model on BT and AT data, adjusted for subject variability. AT, after treatment; BT, before treatment; CB, control beverage; L, the model was built using the log10-transformed variable and the values are back-transformed in the original scale for the reader's convenience; *n*, number of observations; *N*, number of subjects; WA, whole apple.

### Vascular function, blood pressure, arterial stiffness and endothelial function markers

The response to endothelium-dependent microvascular vasodilation (acetylcholine) was greater after the WA intake compared with the CB (*P* = 0.037). There was no effect on blood pressure or other indexes calculated from peripheral and central pulse waveforms (PWA) (**Table 5**, Supplemental Table 7). WA consumption reduced plasma ICAM-1 (*P* = 0.028) compared with the CB (Table 2, Supplemental Table 4). No differences were observed for the other adhesion molecules (Table 2, Supplemental Table 4).

**TABLE 5:** Vascular function, blood pressure, and PWA indexes in mildly hypercholesterolemic participants BT (week 1) and AT (week 8) with WAs or CB^1^

			WAs		CB		
Parameters	L	*n*/*N*	BT (95% CI)	AT (95% CI)	BT (95% CI)	AT (95% CI)	*P* value
LDI							
Ach AUC, PU	✓	132/33	821 (742.5, 908.8)	853 (771.0, 943.6)	784 (708.6, 867.3)	760 (686.9, 840.7)	0.037
SNP AUC, PU	✓	132/33	881 (790.5, 982.6)	911 (816.8, 1015.3)	853 (765.5, 951.5)	829 (743.1, 923.8)	0.135
ABPM							
PP, mm Hg	✓	160/40	42 (39.5, 44.6)	41 (38.5, 43.4)	42 (39.4, 44.4)	41 (38.9, 43.8)	0.699
Brachial SBP, mm Hg	✓	160/40	121 (117.0, 125.0)	120 (116.4, 124.3)	121 (117.0, 125.0)	122 (117.7, 125.7)	0.167
Brachial DBP, mm Hg		160/40	79 (76.1, 81.3)	79 (76.7, 82.0)	79 (76.5, 81.7)	80 (77.6, 82.9)	0.367
PWA							
Central SBP, mm Hg	✓	148/37	114 (109.6, 119.6)	113 (108.7, 118.6)	115 (110.5, 120.5)	115 (109.8, 119.8)	0.388
Central DBP, mm Hg		148/37	76 (73.2, 78.7)	75 (72.2, 77.7)	76 (73.2, 78.7)	75 (72.6, 78.0)	0.700
Central PP, mm Hg	✓	148/37	38 (35.1, 41.5)	38 (35.1, 41.6)	39 (36.1, 42.7)	39 (36.2, 42.8)	0.168
Central MP, mm Hg		148/37	93 (89.7, 96.6)	92 (88.6, 95.5)	94 (90.1, 97.1)	93 (89.4, 96.3)	0.447
Central AP_HR75, %		148/37	6.8 (4.9, 8.6)	7.0 (5.2, 8.9)	7.3 (5.4, 9.2)	7.2 (5.3, 9.1)	0.642
Central AGPH, AS %		148/37	24.8 (20.9, 28.6)	25.4 (21.6, 29.3)	25.9 (22.0, 29.7)	26.5 (22.6, 30.3)	0.217
Central AGPH_HR75, AS %		148/37	16.4 (12.4, 20.3)	17.6 (13.6, 21.5)	18.0 (14.1, 21.9)	18.4 (14.5, 22.4)	0.306
Heart rate, beats/min	✓	148/37	20 (16.2, 24.7)	22 (18.1, 27.6)	23 (18.3, 28.0)	23 (19.0, 29.1)	0.451
Ejection duration, ms		146/37	345.6 (340.5, 350.6)	345.6 (340.5, 350.6)	348.3 (343.2, 353.4)	346.5 (341.4, 351.6)	0.592

1 Values are means (for nontransformed variables mean is equal to median) or medians (for the log-transformed variables) with (500) bootstrap 95% CIs estimated with a joint mixed-effect linear model on BT and AT data, adjusted for subject variability. The treatment effect is statistically significant when *P* < 0.05. ABPM, ambulatory blood pressure monitor; Ach, acetylcholine; AGPH, augmentation pressure/pulse height; AGPH_HR75, heart rate–corrected central augmentation pressure/pulse height; AP_HR75, heart rate–corrected augmented pressure; AS, arterial stiffness; AT, after treatment; BT, before treatment; CB, control beverage; DBP, diastolic blood pressure; L, the model was built using the log10-transformed variable and the values are back-transformed in the original scale for the reader's convenience; LDI, laser Doppler iontophoresis; MP, mean pressure; ms, milliseconds; *n*, number of observations; *N*, number of subjects; PP, pulse pressure; PU, perfusion units; PWA, pulse wave analysis; SBP, systolic blood pressure; SNP, sodium nitroprusside; WA, whole apple.

### Exploratory association between TC response and BA changes

Our initial exploratory analysis showed that WA consumption reduced TC compared with the CB, but also suggested that the level of response to the intervention could be different for men and women (**Supplemental Figures 2–4**). Although the study was not designed to test this hypothesis, exploratory analysis was performed which involved running our full statistical model for TC (primary outcome) separately for the 2 sexes, in order to provide new, even if not conclusive, evidence. TC was reduced after WA intake only in women (treatment effect: −0.33; CI: −0.53, −0.12; *P* = 0.002).

Furthermore, a univariate mixed linear model, taking into consideration the volunteers’ age in addition to the study design, confirmed the link between TC and circulating BAs [lithocholic acid (LCA) and GUDCA] (**Supplemental Table 8**; coefficient values represented in **Supplemental Figure 5**). In particular, the model showed that WA intake produced, on average, a significant decrease in TC and, given that the TC concentration increased with age, the apple intake attenuated this effect. Moreover, the increase in LCA was associated with an increase in TC and, after WA consumption, TC decreased as GUDCA increased. Similar results were obtained with other hydrophilic BAs including glycocholic acid and taurocholic acid (data not shown).

## Discussion

In the present randomized controlled trial, of 40 men and women with moderately raised serum TC, consumption of 2 fresh WAs (Renetta Canada, 340 g) daily decreased serum TC, LDL cholesterol, and plasma ICAM-1 with no detrimental effect on HDL-cholesterol concentrations compared with a sugar-matched control. Few human intervention studies have focused specifically on fresh apples (11–16). Two early studies reported that 2–3 apples/d decreased TC in moderately hypercholesterolemic participants from baseline, although there was no control (9, 10). Later studies in normocholesterolemic (11), hypercholesterolemic (12), and elderly subjects (13) showed no effects, or reported adverse effects on blood lipids (14). A borderline significant decrease in serum TC and LDL cholesterol was found after consumption of fresh WAs (550 g/d) compared with a control period (15). More recently, and in support of our results, TC and LDL cholesterol decreased after the daily consumption of 2 Annurca apples (200 g/d) (16). The potential ability of apples to reduce serum TC has been attributed mainly to the fiber fraction, pectin in particular; however, polyphenols may also play an important role (5). Pure apple polyphenols at daily doses between 600 (23) and 1500 mg (22) decreased TC and LDL cholesterol. In contrast, a cloudy apple juice (803 mg polyphenols) had no effects on blood lipids (20). A total of 990 mg polyphenols (854 mg oligomeric PAs) per day was provided by 2 Renetta Canada apples in our study, which supports the hypothesis that polyphenols play a role in cholesterol lowering. Furthermore, 6 g pectin/d has been reported to lower blood cholesterol according to the European Food Safety Authority (40). The daily intake of soluble fiber in the apple group in our study was 3.7 g. Pectin is the major component of soluble fiber in apples, therefore this amount might not be sufficient alone to lower TC and a major role of polyphenols, especially PAs, and/or a synergetic effect between apple polyphenols and pectin/soluble fiber cannot be excluded (41).

The matrix of a whole food, such as apples, can affect the release of nutrients, e.g., sugars, fiber, and polyphenols, as well as their fate and function in the body compared with a simple sugar beverage (5). Of the aforementioned studies, two have explored the effects of a clear apple juice on lipid metabolism in comparison with WAs. Ravn-Haren et al. (15) have shown that a clear apple juice (total polyphenols, 108 mg/d; total pectin, 0.03 g/d; total sugars, 63 g/d) intake for 4 wk, in 23 healthy participants, increased LDL cholesterol compared with WAs (total polyphenols, 239 mg/d; total pectin, 2.87 g/d; total sugars, 51 g/d). On the contrary, Hyson et al. (11) found no differences in blood lipid concentrations of 25 healthy participants after the intake of fresh WAs or an unsupplemented apple juice for 6 wk; however, this study did not include nutrition information about the tested products. In our study, although there was a tendency toward a significant increase in insulin after the CB, changes in glucose and adiponectin were not seen. However, potential further adverse effects on lipid and glucose metabolism related to the food matrix and glycemic index of the CB compared with the WA cannot be excluded.

Endothelial function can serve as an indicator of cardiovascular health. We found small but significant improvements in endothelium-dependent microvascular vasodilation (42) after the apple intake. To date, only 2 studies to our knowledge have assessed the long-term effects of apple intake on vascular function, showing beneficial effects with high-flavonoid apples (19) but not with dried apples (17). Improvement in vascular function has been mainly shown with a high daily dose of flavonoid monomers such as epicatechin (48–180 mg) and/or quercetin (184–195 mg), either postprandially (19, 24) or after a 4-wk treatment (19). The lower daily intakes of flavanol monomers in our study suggest a potential role for the apple PAs. Longer-term studies with PA-rich foods/beverages such as chocolate (43) and tea (44) have also reported beneficial effects on vascular function. Although PA bioavailability is low compared with the readily absorbed flavanol monomers, PAs with a degree of polymerization >4 reach the colon almost intact where they are degraded into phenolic acids by gut bacteria and absorbed into the circulation, where they may contribute to systemic health-promoting properties (45).

In addition, endothelial dysfunction has been related to a proinflammatory phenotype. Endothelial adhesion molecules such as VCAM-1, ICAM-1, E-selectin, and P-selectin are proinflammatory proteins which play a critical role in the adhesion of leukocytes to endothelial cells during the early stages of atherosclerosis (46). Their concentration is low in normal conditions, but they can increase when the endothelium is activated by stimuli such as proinflammatory cytokines and reactive oxygen species (46). In the present study, apple intake reduced plasma ICAM-1. ICAM-1 and VCAM-1 have been shown to decrease after a vitamin C–rich apple juice (21), but not after the intake of a cloudy apple juice (20). Nevertheless, PAs from other sources such as red wine (47) have also been shown to decrease ICAM-1 and VCAM-1 in healthy humans.

Circulating BAs have been linked with regulatory functions in lipid and energy metabolism via binding to nuclear receptors and acting as signaling molecules (48). In our study, no differences were observed in blood BA concentrations. However, BA metabolism is complex and regulated by many parameters, which are still unclear (49). Interestingly, our exploratory analysis indicated differential TC and BA profile responses in men and women after apple consumption, with circulating LCA and GUDCA predicting the TC response in women. LCA is an agonist of farnesoid X receptor (FXR), activation of which reduces BA synthesis in the liver via fibroblast growth factor-19 signaling (50, 51), whereas GUDCA is an antagonist for FXR, recently shown to improve metabolic endpoints in humans related to diabetes (52). Significant sex differences in TC response after apple intake have been reported previously, with women showing the largest response (15). Similar sex differences have been shown to be dependent on BAs and their receptors in animal studies (53, 54). However, our study was not powered to explore sex differences; therefore, this warrants closer examination in specifically designed human intervention studies powered for comparison between sexes.

Strengths of this study include its adequately powered sample size, duration, crossover design, the use of a sugar- and energy-matched control, and the determination of a wide range of outcomes relative to other similar studies focusing on fresh apples (5). Another strength is the confirmation of good compliance using appropriate recognized biomarkers of apple intake in urine (phloretin metabolites). However, there were some limitations. The volunteers’ diet was not restricted in terms of polyphenol-rich products such as fruits, vegetables, and beverages as in other similar studies (15). However, polyphenols and fiber dietary intake increased only after the intake of WAs and compliance to the treatments was confirmed. Another potential limitation was that volunteers were not blinded to the interventions, a common limitation when using whole foods (fresh apples). However, participants were told there may be benefit from either intervention and researchers in the study were blinded during analysis. In addition, the use of an apple beverage as a control, although appropriate to explore the potential beneficial effects of the polyphenol and fiber components of a whole food such as apple, does not take into account the effects of the food matrix as already discussed. Finally, this study was not powered to explore sex differences; these are only exploratory and need confirmation in studies adequately powered to explore sex effects.

In conclusion, our findings show clear cause and effect between inclusion of 2 Renetta Canada apples into normal diets and improved CVD risk factors, by reducing TC, LDL cholesterol, and ICAM-1 and increasing microvascular vasodilation, in healthy subjects with mildly raised serum cholesterol concentrations. Dose- and structure-response studies are necessary to explore the potential mechanisms, which are likely to involve BA signaling and/or small phenolic acids derived from apple polyphenols, both linked to gut microbiota metabolism.

## Supplementary Material

nqz282_Supplemental_File

## Notes

Supported in part by AGER (Agribusiness and research) grant no. 2010-2119 funding the project “Apple fruit quality in the post-genomic era, from breeding new genotypes to post-harvest: nutrition and health,” Fondazione Edmund Mach internal funding, and European Union Horizon2020 research and innovation grant agreement no. 696295 (to KMT)—ERA-Net Cofund ERA-HDHL “Biomarkers for Nutrition and Health implementing the JPI HDHL objectives” (https://www.healthydietforhealthylife.eu/), projects FOODBALL (http://foodmetabolome.org/) and CABALA_diet&health (http://www.cabalaproject.eu/). The test apples were kindly provided by Consorzio Melinda SCA, Cles, Trentino, Italy.

Data described in the article will be made available upon request pending application and approval.

Supplemental Methods, Supplemental Tables 1–8, and Supplemental Figures 1–5 are available from the “Supplementary data” link in the online posting of the article and from the same link in the online table of contents at https://academic.oup.com/ajcn/.

Abbreviations used: AIx, Augmentation Index; BA, bile acid; C-AGPH, central augmentation pressure-to-pulse height ratio; CB, control beverage; CVD, cardiovascular disease; FXR, farnesoid X receptor; GUDCA, glycoursodeoxycholic acid; HR, heart rate; ICAM-1, intercellular cell adhesion molecule-1; LDI, laser Doppler imaging; LH, lithium heparin; NEFA, nonesterified fatty acid; PA, proanthocyanidin; PU, perfusion units; PWA, pulse wave analysis; SST, serum separator tube; TAG, triacylglycerol; TC, total cholesterol; VCAM-1, vascular cell adhesion molecule-1; WA, whole apple.
