# Supplementary material for: Two apples a day lower serum cholesterol and improve cardiometabolic biomarkers in mildly hypercholesterolemic adults: a randomized, controlled, crossover trial
Source: Am J Clin Nutr. 2019 Dec 16;111(2):307–18. doi: 10.1093/ajcn/nqz282 (PMC6997084; doi:10.1093/ajcn/nqz282)
Supplement: nqz282_Supplemental_File [file nqz282_supplemental_file.pdf]

Two apples a day lower serum cholesterol and improve cardiometabolic biomarkers in mildly hypercholesterolemic adults: a randomized, controlled, crossover trial
by Koutsos et al. (2019)
Online Supplementary Material

Determination of bile acids in blood

Reagents

HPLC-grade methanol, acetonitrile, 2-propanol, acetone and formic acid were obtained from Sigma Aldrich. The ultrapure water was obtained by purifying demineralized water in a Milli-Q system from Millipore (Bedford, MA, USA). Internal deuterated standards: cholic acid-d4, lithocholic acid-d4, deoxycholic acid-d4, chenodeoxycholic acid-d4, ursodeoxycholic acid-d4, glycocholic acid-d4, glycolithocholic acid-d4, glycodeoxycholic acid-d4, glycochenodeoxycholic acid-d4, were obtained from Chemical Research 2000 S.r.l; while native standards: cholic acid, lithocholic acid, deoxycholic acid, chenodeoxycholic acid, ursodeoxycholic acid, glycocholic acid, glycolithocholic acid, glycodeoxycholic acid, glycochenodeoxycholic acid, glycoursodeoxycholic acid, taurocholic acid, taurolithocholic acid, taurodeoxycholic acid, taurochenodeoxycholic, tauroursodeoxycholic were supplied by Sigma Aldrich. Sirocco protein precipitation plate from Waters, (USA).

Sample preparation

Plasma samples dedicated bile acids quantification method were prepared with Sirocco protein precipitation plate (Waters, USA), as described elsewhere (Trost et al., 2018). Briefly, 100 µL of heparin plasma was thawed on ice and placed in a Sirocco with 200 µL of internal standards dissolved in methanol and 200 µL of solvent consisting of 0.1 % formic acid in methanol: water (4:1 V/V). Samples were filtered using positive pressure-96 manifold (Waters, USA), followed by elution with 400 ul of solvent acetonitrile: acetone (4:1 V/V). Samples were evaporated to dryness using a Techne Dr-block DB 3D heater at room temperature and redissolved with 200 ml of water: methanol (1:1 V/V). Additionally, pool plasma sample (QC) was prepared consisting of 20 uL of each plasma.

Instrumental analysis

Chromatographic separation of the compounds was made using the Kinetex C18 column (150 mm × 2.1 mm I.D., particle size 3.5 µm) with pre-column 4.0 mm x 2.0 mm I.D (Phenomenex Torrance, CA, USA). A triple quadrupole mass spectrometer system (5500 Triple Quad AB Sciex Instruments, Foster City, CA, USA) with an electrospray ionization (ESI) source coupled to an

Dionex UHPLC system was used for analysis of metabolites. Mobile phases A consisted of acetonitrile 40% in water, NH4COOH 10 mM and HCOOH 0.1% while phase B consisted of isopropanol 90%, acetonitrile 10%, NH4COOH 10mM and HCOOH 0.1%.
A linear gradient was set from 5% of A for 2 min, to 100% of A in 20 min, and maintained at 100% of A for 5 min. The flow rate was 400 μL/min. The injection volume was 20 μL.
The ESI source was operated in polarity switching mode and the parameter settings were: the source temperature was set at 250 °C, the nebulizer gas (Gas1) and heater gas (Gas2) at 40 and 20 psi respectively (1psi = 6894.76 Pa). UHPLC nitrogen (99.999%) was used as both curtain and collision gas(CAD) at 20 and 9 psi respectively. Nitrogen was used as the nebulizer gas, curtain gas and collision gas. The two most abundant fragments to use as quantifier and qualifier were identified for each compound. Declustering potential (DP) and entrance potential (EP) were optimized for each precursor ion and collision energy (CE) and Collision Cell Exit Potential (CXP) for each product ion by direct infusion of analytical standards. Table 1a shows the compound-specific instrumental parameters used in the analytical method. Once mass spectrometer parameters were defined for all compounds of our interest, QC sample and analytical standards were injected with LC column in unscheduled mode to obtain retention time information. Table S1a shows all mass spectrometer parameters and retention time for all compounds considered in this method.

Method Validation

The method validation assays were performed according to the currently accepted US Food and Drug Administration (FDA) bio- analytical method validation guide (US CDER, 2001). Validation assays were established on calibration standards and quality control (QC) samples prepared as a pool of plasma samples. The linearity in the analytical response, the limits of detection and quantification (LODs and LOQs, respectively) were determined using a mix of standards while repeatability was done with QC pooled samples (N=42) injected during the sequence. To assess linearity solvent and matrix-matched calibration curves were prepared by spiking plasma extracts at different concentration levels. The degree of enhancement or suppression due to the matrix effect was calculated as follows: ((solvent slope)/(matrix slope)*100 – 100) (Table S2b). Assessment of recoveries was done with use of deuterated standards added at three concentrations levels into QC plasma samples: ca 0.2 uM, ca 2.4 uM and ca 24 uM (See Table S1c for details), different concentrations levels reflects variability of concentrations found in real samples, each concentration level was repeated 3 times.

Supplemental Table 1. Summary of tandem mass spectrometry parameters used for multiple reaction monitoring (MRM) detection of the bile acids: molecular weight, retention time, mode of ionization, selected precursor ion, and selected product ions (Q1, qualifier; Q2, quantifier; DP: declustering potential; EP: entrance potential; CEP: collision cell entrance potential; CE: collision energy; CXP: collision cell exit potential).

Compounds	Abbreviation	MW	Rt (min) 30min	Ionization Mode	Precursor ion	Q1 Product Ion	DP	EP	CE	CXP	Q2 Product Ion	DP	EP	CE	CXP
**NATIVE COMPOUNDS:**															
Cholic acid	CA	408.5	13.8	$[M+HCOO]^-$	453.2	407.3	-125	-10	-30	-29	345.3	-300	-10	-42	-23
Lithocholic acid	LCA	375.6	17.0	$[M+HCOO]^-$	421.2	375.3	-60	-10	-32	-29	44.9	-60	-10	-56	-21
Deoxycholic acid	DCA	392.6	15.5	$[M+HCOO]^-$	437.2	391.4	-70	-10	-30	-23	345.2	-205	-10	-46	-21
Chenodeoxycholic acid	CDCA	392.6	15.2	$[M+HCOO]^-$	437.2	391.3	-85	-10	-32	-25	45.0	-85	-10	-74	-7
Ursodeoxycholic acid	UDCA	392.6	13.8	$[M+HCOO]^-$	437.2	391.3	-65	-10	-30	-31	45.0	-65	-10	-64	-7
Glycocholic acid	G-CA	465.6	12.3	[M-H]-	464.2	74.1	-220	-10	-78	-9	402.3	-220	-10	-48	-25
Glycolithocholic acid	G-LCA	433.6	15.1	[M-H]-	432.2	74.0	-235	-10	-80	-11	388.3	-235	-10	-46	-31
Glycodeoxycholic acid	G-DCA	449.6	13.9	[M-H]-	448.2	74.1	-220	-10	-78	-9	404.3	-220	-10	-46	-25
Glycochenodeoxycholic acid	G-CDCA	449.6	13.6	[M-H]-	448.2	74.0	-230	-10	-70	-9	386.3	-230	-10	-48	-25
Glycoursodeoxycholic acid	G-UDCA	449.6	12.1	[M-H]-	448.2	74.1	-225	-10	-72	-9	386.3	-225	-10	-48	-25
Taurocholic acid	T-CA	515.6	11.5	[M-H]-	514.2	80.0	-200	-10	-128	-9	124.1	-200	-10	-68	-11
Taurolithocholic acid	T-LCA	483.6	13.9	[M-H]-	482.2	80.0	-280	-10	-130	-13	124.1	-280	-10	-66	-9
Taurodeoxycholic acid	T-DCA	499.6	12.9	[M-H]-	498.2	80.1	-300	-10	-130	-9	124.1	-300	-10	-66	-9
Taurochenodeoxycholic	T-CDCA	499.6	12.5	[M-H]-	498.2	80.0	-290	-10	-130	-9	124.1	-290	-10	-66	-11
Tauroursodeoxycholic	T-UDCA	499.6	11.2	[M-H]-	498.2	80.0	-275	-10	-130	-9	124.1	-275	-10	-64	-11
**LABELED COMPOUNDS:**															
Cholic acid-d_4	CA-d_4	412.6	13.9	$[M+HCOO]^-$	457.2	411.3	-95	-10	-38	-33	347.3	-235	-10	-46	-25
Lithocholic acid-d_4	LCA-d_4	380.6	17.0	$[M+HCOO]^-$	425.2	379.3	-75	-10	-32	-27	45.1	-75	-10	-64	-7
Deoxycholic acid-d_4	DCA-d_4	396.6	15.6	$[M+HCOO]^-$	441.1	395.3	-80	-10	-32	-25	349.3	-235	-10	-46	-19
Chenodeoxycholic acid-d_4	CDCA-d_4	396.6	15.3	$[M+HCOO]^-$	441.2	395.3	-75	-10	-32	-23	45.0	-75	-10	-60	-7
Ursodeoxycholic acid-d_4	UDCA-d_4	453.8	13.8	$[M+HCOO]^-$	441.2	395.3	-100	-10	-32	-23	45.1	-100	-10	-58	-7
Glycocholic acid-d_4	G-CA-d_4	469.6	12.4	[M-H]-	468.2	406.3	-200	-10	-50	-23	74.1	-200	-10	-84	-7
Glycolithocholic acid-d_4	G-LCA-d_4	437.6	15.2	[M-H]-	436.2	392.4	-165	-10	-46	-23	74.0	-165	-10	-74	-9
Glycodeoxycholic acid-d_4	G-CDCA-d_4	453.6	13.7	[M-H]-	452.2	390.3	-180	-10	-50	-23	74.0	-180	-10	-78	-7
Glycochenodeoxycholic acid-d_4	G-UDCA-d_4	453.6	12.1	[M-H]-	452.2	390.3	-185	-10	-50	-23	74.0	-185	-10	-74	-7

Online Supplementary Material

Supplemental Table 2. Characteristics of the method for the analysis of Bile Acids, respectively in solvent and in matrix, by Ultra-High Performance Liquid Chromatography Electrospray Ionization Tandem Mass Spectrometry (UHPLC–ESI-MS/MS) tandem mass spectrometry parameters. Limit of quantification, limit of detection, linearity range and matrix effect and repeatability.

**Compounds**	**Abbr.**	**LOQ (uM)**	**LOD (uM)**	**Upper Linearity range (uM)**	**Solvent Calibration Curve**	**R^2**	**Qt/Qn**	**Stdev (Qt/Qn)**	**Linearity Range Matrix uM**		**Matrix Calibration Curve**	**R^2**	**Matrix Effect**	**Repeatability Pooled QC N=42 (RSD %)**
Cholic acid	CA	0.0023	0.0005	4.52	y = 17882981x +916	1.000	7.17	0.59	0.003	3.01	y = 114664044x +167	0.999	18.0	10.2
Lithocholic acid	LCA	0.0009	0.0003	3.79	y = 16659374x -156507	0.995	3.18	0.14	0.003	3.01	y = 14778531x +33875	1.000	11.3	11.0
Deoxycholic acid	DCA	0.0012	0.0003	4.61	y = 24172333x -240189	0.997	3.26	0.13	0.003	3.07	y = 22433931x +202187	0.999	7.2	11.0
Chenodeoxycholic acid	CDCA	0.0012	0.0004	4.72	y = 18179975x +3568	0.999	5.17	0.28	0.003	3.01	y = 15391389x -112512	0.999	15.3	6.7
Ursodeoxycholic acid	UDCA	0.0027	0.0009	3.59	y = 19238539x -71131	1.000	4.95	0.25	0.003	2.94	y = 17336186x +60943	0.998	9.9	8.4
Glycocholic acid	G-CA	0.0027	0.0005	5.37	y = 7343987x +12674	1.000	3.41	0.14	0.003	3.02	y = 6596448x -84390	0.999	10.2	9.9
Glycolithocholic acid	G-LCA	0.0011	0.0003	4.38	y = 11158361x -165831	0.995	3.43	0.16	0.003	2.92	y = 9505642x -27006	1.000	14.8	13.6
Glycodeoxycholic acid	G-DCA	0.0011	0.0003	4.27	y = 8904598x -41141	0.999	3.74	0.45	0.003	3.02	y = 9993836x -124808	0.999	-12.2	8.0
Glycochenodeoxycholic acid	G-CDCA	0.0013	0.0004	5.17	y = 8338832x -46732	0.999	3.94	0.26	0.003	3.04	y = 8033235x -346997	0.999	3.7	8.3
Glycoursodeoxycholic acid	G-UDCA	0.0011	0.0003	4.43	y = 9840977x -68520	0.999	4.84	0.22	0.003	2.94	y = 8545156x -69270	0.999	13.2	5.8
Taurocholic acid	T-CA	0.0005	0.0003	5.17	y = 12562772x +69893	1.000	4.96	0.16	0.003	2.95	y = 13051406x -8673	1.000	-3.9	5.3
Taurolithocholic acid	T-LCA	0.0004	0.0002	4.36	y = 16037995x +75642	1.000	6.73	0.44	0.003	3.03	y = 18878039x +16915	1.000	-17.7	7.3
Taurodeoxycholic acid	T-DCA	0.0004	0.0001	4.69	y = 17217820x +101408	1.000	4.75	0.21	0.003	3.00	y = 18895568x +37414	0.999	-9.7	15.5
Taurochenodeoxycholic	T-CDCA	0.0004	0.0002	4.25	y = 14710182x +107441	1.000	6.79	0.37	0.003	3.00	y = 15599807x -29635	0.999	-6.0	9.6
Tauroursodeoxycholic	T-UDCA	0.0004	0.0002	4.41	y = 15311991x -146115	0.999	6.74	0.65	0.003	2.92	y = 17030929x +1897	0.999	-11.2	/

Supplemental Table 3. Assessment of the recovery in plasma estimated from the analysis of deuterated standard of Bile Acids.

CA_D4		LCA_D4		DCA_D4		CDCA_D4		UDCA_D4		GCA_D4		GLCA_D4		GCDCA_D4		GUDCA_D4	
uM	%	uM	%	uM	%	uM	%	uM	%	uM	%	uM	%	uM	%	uM	%
0.24	101.1	0.26	86.3	0.25	86.2	0.25	73.1	0.25	110.9	0.21	93.8	0.23	95.4	0.22	101.7	0.22	89.8
2.42	86.8	2.63	82.0	2.52	85.0	2.52	82.9	2.52	76.8	2.13	77.9	2.28	79.7	2.20	78.5	2.20	75.2
24.24	96.7	26.27	106.4	25.21	101.2	25.21	104.6	25.21	91.8	21.29	91.8	22.85	93.6	22.04	89.8	22.04	103.6

Determination of phloretin metabolites in urine

Untargeted assay of urine was performed with use a hybrid linear ion trap Fourier Transform (LTQ FT) Orbitrap mass spectrometer (Thermo Fisher, Bremen, Germany) interfaced to a Dionex HPLC system, consisting of auto sampler and quaternary gradient HPLC-pump. Method was published elsewhere (1). The Orbitrap LTQ was equipped with an Electrospray Ionization (ESI) probe and operated in both positive and negative ionization modes. Each sample was analysed, both under positive and negative ionization, using two different mass acquisition methods for each ionization mode. The first method consisted of a full scan (mass range from 100 to 1000 Da) at a mass resolution of 30,000 FWHM (full width at half maximum for m/z 400) in centroid mode. Then, based on the data dependent acquisition (DDA) mode, the LC-MS/MS analysis of each sample was performed in order to achieve the mass fragmentation spectra. In this method during the chromatographic run, both full scan and MS^2 spectra of the 3 most intense ions of each full scan were acquired. The resolving power for MS^2 scans was 7500. Product ions were generated in the LTQ trap at collision energy 35 eV using an isolation width of 1 Da.

Chromatographic separation of the compounds was made using the Kinetex C18 column (150 mm × 2.1 mm I.D., particle size 3.5 µm) with pre-column 4.0 mm x 2.0 mm I.D (Phenomenex Torrance, CA, USA). Following gradient was used at a constant flow rate of 0.3 mL /min using Milli-Q water (Solvent A) and acetonitrile (Solvent B) both with 0.1% formic acid. The composition of mobile phases was: 95 % of solvent A and 5 % of solvent B were maintained for one minute, followed by an increase of solvent B to 45 % in 12 min and to 80 % in 2 min and maintained for 2 min. The initial composition was restored in 3 min. Two metabolites of phloretin were found: phloretin sulfate and phloretin glucuronide sulfate at *m/z* 449.1051 (-) and *m/z* 529.0621 (-) respectively. Analysis of high resolution MS/MS spectra allowed for identification at level II of both metabolites, by comparison to original analytical standard of phloretin after loss of glucuronide and sulfate moieties. Data were used for verification of compliance to the diet.

Online Supplementary Material

References

1. Ulaszewska MM, Trost K, Stanstrup J, Tuohy KM, Franceschi P, Chong MF, George T, Minihane AM, Lovegrove JA, Mattivi F. Urinary metabolomic profiling to identify biomarkers of a flavonoid-rich and flavonoid-poor fruits and vegetables diet in adults: the FLAVURS trial. Metabolomics. 2016;12(2):32.

Supplemental Table 4. Anthropometric characteristics and blood biochemistry in mildly hypercholesterolemic participants before (BT) (week 1) and after (AT) treatment (week 8) with whole apples or control beverage - details of the mixed linear model [1].

Parameters	L	n/N	Sequence Effect (95% CI)	Period Effect (95% CI)	Treatment Effect (95% CI)	R^2	Ω^2
Anthropometrics							
Weight (kg)		160/40	-3.15 (-12.34,5.96)	0.06 (-0.29,0.43)	-0.10 (-0.58,0.41)	1.00	1.00
BMI (kg/m2)		160/40	-1.56 (-3.98,0.82)	0.00 (-0.12,0.13)	-0.06 (-0.22,0.12)	0.99	0.99
Waist (cm)		144/39	-4.95 (-12.23,2.06)	1.20 (0.43,2.05)	0.07 (-1.09,1.10)	0.97	0.97
Total Body Fat (%)		152/40	-4.76 (-9.50,0.11)	-0.04 (-0.42,0.37)	0.20 (-0.35,0.72)	0.98	0.98
Trunk Fat (%)		150/40	-4.29 (-8.55,-0.05)	-0.13 (-0.67,0.40)	0.39 (-0.35,1.06)	0.96	0.96
Blood biochemistry							
Total Cholesterol (mmol/l)		152/38	-0.51 (-0.93,-0.03)	0.07 (-0.03,0.18)	-0.22 (-0.37,-0.07)	0.87	0.87
LDL-Cholesterol (mmol/l)		152/38	-0.33 (-0.71,0.11)	0.06 (-0.02,0.16)	-0.14 (-0.26,-0.02)	0.88	0.88
HDL-Cholesterol (mmol/l)		152/38	0.01 (-0.22,0.26)	0.03 (0.00,0.06)	-0.03 (-0.07,0.02)	0.95	0.95
TAG (mmol/l)	✓	152/38	-0.14 (-0.23,-0.05)	-0.02 (-0.04,0.01)	-0.05 (-0.08,-0.01)	0.82	0.82
NEFA (µmol/l)	✓	152/38	-0.05 (-0.14,0.05)	0.07 (0.04,0.10)	0.01 (-0.04,0.05)	0.77	0.77
Glucose (mmol/)		152/38	0.04 (-0.21,0.31)	-0.06 (-0.12,0.01)	-0.06 (-0.15,0.03)	0.84	0.84
Insulin (pmol/l)	✓	160/40	-0.15 (-0.27,-0.02)	0.01 (-0.03,0.05)	-0.05 (-0.10,0.00)	0.80	0.80

TNF-alpha (pg/ml)		160/40	-2.60 (-4.12,-1.09)	0.01 (-0.36,0.39)	-0.41 (-0.90,0.12)	0.88	0.88
Albumin (g/l)		152/38	-0.14 (-1.48,1.28)	0.81 (0.40,1.28)	-0.44 (-1.08,0.18)	0.77	0.77
Adiponectin (µg/ml)	✓	152/38	-0.06 (-0.27,0.13)	0.00 (-0.02,0.02)	0.02 (0.00,0.05)	0.97	0.97
Endothelin (pg/ml)	✓	160/40	-0.01 (-0.08,0.06)	-0.01 (-0.04,0.02)	0.01 (-0.02,0.05)	0.71	0.71
Uric acid (µmol/l)		152/38	5.76 (-35.38,53.18)	2.86 (-3.28,9.90)	11.34 (1.80,20.51)	0.93	0.93
TAC (mmolL TroloxEquiv)		152/38	0.05 (-0.08,0.18)	-0.02 (-0.05,0.01)	0.04 (-0.01,0.08)	0.85	0.85
VCAM-1 (ng/ml)	✓	152/38	0.06 (-0.01,0.13)	0.01 (-0.01,0.02)	-0.02 (-0.03,0.00)	0.91	0.91
ICAM-1 (ng/ml)	✓	152/38	0.02 (-0.09,0.14)	0.00 (-0.01,0.01)	-0.02 (-0.03,0.00)	0.98	0.98
E selectin (ng/ml)	✓	152/38	-0.08 (-0.17,0.01)	0.00 (-0.01,0.02)	-0.01 (-0.02,0.01)	0.96	0.96
P selectin (ng/ml)	✓	152/38	0.00 (-0.06,0.06)	0.01 (0.00,0.02)	-0.01 (-0.02,0.00)	0.95	0.95

[1] The values are the effect estimate obtained with a joint mixed model computed on BT and AT data, adjusted for subject variability, and are presented with 95% bootstrap confidence interval (with 500 replicates). The significance of the observed effect is evaluated both in a data-driven fashion (as treatment effect with 95% CIs) and with a classical statistic approach (using the p-value). The effect is statistically significant when the CIs do not include zero or when P<0.05; L: the model was built using the variable after log10-transformation; n: number of observation; N: number of subjects; ICAM: intercellular-CAM-1; NEFA: non esterified fatty acids; TAC: total antioxidant capacity; TAG: triacylglycerol; TNF: tumor necrosis factor; VCAM-1: vascular cell adhesion molecule-1.

Supplemental Table 5. Dietary nutrient intake in mildly hypercholesterolemic participants before (BT) (week 1) and after (AT) treatment (week 8) with whole apples or control beverage - details of the mixed linear model [1].

Parameters	T	n/N	Sequence Effect (95% CI)	Period Effect (95% CI)	Treatment Effect (95% CI)	R^2
Energy (KJ)	L	156/39	-0.02 (-0.10,0.06)	0.01 (-0.01,0.03)	0.01 (-0.02,0.03)	0.824
Energy (kcal)	L	156/39	-0.02 (-0.10,0.06)	0.01 (-0.01,0.03)	0.00 (-0.02,0.03)	0.824
Carbohydrates (g)		156/39	-14.20 (-51.00,21.40)	14.54 (2.26,26.85)	12.55 (-3.58,29.70)	0.675
Total sugar (g)		156/39	1.20 (-19.79,21.06)	6.35 (-2.58,15.31)	-2.65 (-14.38,9.83)	0.524
Fat (g)	L	156/39	-0.01 (-0.11,0.09)	0.00 (-0.03,0.03)	0.00 (-0.04,0.04)	0.745
Protein (g)	L	156/39	0.01 (-0.06,0.08)	0.01 (-0.02,0.03)	0.01 (-0.02,0.04)	0.623
Fiber (AOAC) (g)		156/39	-3.47 (-7.54,0.48)	1.27 (-0.09,2.64)	6.62 (4.83,8.52)	0.714
Total flavonoids (mg)	S	156/39	-1.51 (-8.33,5.08)	0.23 (-1.96,2.43)	18.55 (15.67,21.60)	0.760

[1] The values are the effect estimate obtained with a joint mixed model computed on BT and AT data, adjusted for subject variability, and are presented with 95% bootstrap confidence interval (with 500 replicates). The significance of the observed effect is evaluated both in a data-driven fashion (as treatment effect with 95% CIs) and with a classical statistic approach (using the p-value). The effect is statistically significant when the CIs do not include zero or when $P<0.05$; T: the model was built using a transformed version of the variable (L=log10, S=square root) and the values are back-transformed in the original scale for reader's convenience; n: number of observation; N: number of subjects.

Supplemental Table 6. Fasted circulating plasma bile acid (BA) concentrations in mildly hypercholesterolemic participants before (BT) (week 1) and after (AT) treatment (week 8) with whole apples or control beverage - details of the mixed linear model [1].

Parameters	L	n/N	Sequence Effect (95% CI)	Period Effect (95% CI)	Treatment Effect (95% CI)	R^2	Ω^2
Cholic acid (CA) (nM)	✓	160/40	0.32 (0.02,0.61)	-0.02 (-0.13,0.10)	-0.07 (-0.22,0.09)	0.73	0.72
Chenodeoxycholic acid (CDCA) (nM)	✓	160/40	0.27 (-0.03,0.56)	-0.04 (-0.15,0.07)	-0.07 (-0.22,0.09)	0.73	0.72
Deoxycholic acid (DCA) (nM)	✓	160/40	0.17 (-0.08,0.42)	0.00 (-0.08,0.08)	-0.03 (-0.14,0.09)	0.77	0.77
Lithocholic acid (LCA) (nM)	✓	160/40	0.04 (-0.07,0.15)	0.00 (-0.06,0.05)	-0.02 (-0.09,0.05)	0.62	0.59
Glycocholic acid (GCA) (nM)	✓	160/40	0.03 (-0.19,0.25)	0.01 (-0.07,0.10)	0.00 (-0.11,0.12)	0.70	0.69
Glycochenodeoxycholic acid (GCDCA) (nM)	✓	160/40	0.05 (-0.19,0.28)	-0.07 (-0.16,0.02)	0.01 (-0.11,0.13)	0.72	0.70
Glycodeoxycholic acid (GDCA) (nM)	✓	160/40	0.08 (-0.23,0.39)	-0.03 (-0.11,0.06)	0.07 (-0.05,0.19)	0.82	0.81
Glycolithocholic acid (GLCA) (nM)	✓	140/38	-0.02 (-0.17,0.15)	-0.04 (-0.14,0.05)	0.02 (-0.12,0.18)	0.51	0.46
Glycoursodeoxycholic acid (GUDCA) (nM)	✓	160/40	0.16 (-0.16,0.47)	-0.02 (-0.12,0.08)	-0.03 (-0.16,0.11)	0.78	0.78
Taurocholic acid (TCA) (nM)	✓	160/40	0.05 (-0.18,0.27)	0.06 (-0.03,0.15)	0.00 (-0.12,0.13)	0.69	0.67
Taurochenodeoxycholic acid (TCDCA) (nM)	✓	160/40	0.04 (-0.20,0.28)	0.03 (-0.06,0.12)	-0.01 (-0.13,0.11)	0.73	0.72
Taurodeoxycholic.acid (TDCA) (nM)	✓	160/40	0.04 (-0.22,0.30)	0.05 (-0.03,0.14)	0.05 (-0.07,0.17)	0.77	0.76
Taurolithocholic acid (TLCA) (nM)	✓	160/40	-0.01 (-0.09,0.07)	0.03 (-0.02,0.08)	0.04 (-0.02,0.10)	0.52	0.47

[1] The values are the effect estimate obtained with a joint mixed model computed on BT and AT data, adjusted for subject variability, and are presented with 95% bootstrap confidence interval (with 500 replicates). The significance of the observed effect is evaluated both in a data-driven fashion (as treatment effect with

95% CIs) and with a classical statistic approach (using the p-value); The effect is statistically significant when the CIs do not include zero or when $P<0.05$; L: the model was built using the variable after log10-transformation; n: number of observation; N: number of subjects.

Supplemental Table 7. Vascular function, blood pressure and PWA indices in mildly hypercholesterolemic participants before (BT) (week 1) and after (AT) treatment (week 8) with whole apples or control beverage - details of the mixed linear model [1].

Parameters	L	n/N	Sequence Effect (95% CI)	Period Effect (95% CI)	Treatment Effect (95% CI)	R^2	Ω^2
LDI							
Ach AUC (PU)	✓	132/33	0.06 (0.00,0.13)	-0.01 (-0.04,0.02)	0.05 (0.00,0.09)	0.585	0.559
SNP AUC (PU)	✓	132/33	0.05 (-0.02,0.11)	-0.02 (-0.06,0.02)	0.04 (-0.02,0.09)	0.509	0.468
ABPM							
PP (mmHg)	✓	160/40	-0.02 (-0.06,0.02)	0.01 (0.00,0.03)	0.00 (-0.03,0.02)	0.720	0.709
Brachial SBP (mmHg)	✓	160/40	0.00 (-0.03,0.02)	0.00 (0.00,0.01)	0.00 (-0.01,0.00)	0.901	0.900
Brachial DBP (mmHg)		160/40	0.69 (-4.06,5.25)	-0.03 (-1.46,1.47)	-0.89 (-2.81,1.18)	0.785	0.779
PWA							
Central SBP (mmHg)	✓	148/37	-0.01 (-0.05,0.03)	0.01 (0.00,0.01)	0.00 (-0.01,0.01)	0.893	0.891
Central DBP (mmHg)		148/37	-0.10 (-4.91,4.80)	2.37 (1.12,3.58)	-0.35 (-2.23,1.57)	0.827	0.824
Central PP (mmHg)	✓	148/37	-0.03 (-0.10,0.04)	0.00 (-0.02,0.01)	-0.01 (-0.03,0.01)	0.906	0.905
Central MP (mmHg)		148/37	-1.16 (-7.55,5.31)	2.28 (0.87,3.67)	-0.79 (-2.93,1.40)	0.862	0.860
Central AP_HR75 (AS) (%)		148/37	-1.84 (-5.39,1.88)	-0.04 (-0.56,0.46)	-0.18 (-0.96,0.62)	0.937	0.937

Central AGPH (%)		148/37	-3.47 (-10.73,4.08)	-0.58 (-1.70,0.51)	-1.02 (-2.71,0.71)	0.929	0.929
Central AGPH_HR75 (%)		148/37	-4.59 (-12.01,3.13)	-0.40 (-1.52,0.70)	-0.85 (-2.55,0.89)	0.932	0.932
Heart rate (beats/min)	✓	148/37	-0.08 (-0.25,0.09)	-0.01 (-0.05,0.03)	-0.02 (-0.08,0.04)	0.848	0.845
Ejection Duration (ED) (ms)		146/37	3.24 (-5.91,12.76)	-0.85 (-3.22,1.80)	-0.94 (-4.36,2.59)	0.824	0.820

[1] The values are the effect estimate obtained with a joint mixed model computed on BT and AT data, adjusted for subject variability, and are presented with 95% bootstrap confidence interval (with 500 replicates). The significance of the observed effect is evaluated both in a data-driven fashion (as treatment effect with 95% CIs) and with a classical statistic approach (using the p-value); The effect is statistically significant when the CIs do not include zero or when P<0.05; L: the model was built using the variable after log10-transformation; n: number of observations; N: number of subjects; ABPM: ambulatory blood pressure monitor; Ach: acetylcholine; AGPH_HR75: heart rate corrected central augmentation pressure/pulse height; AGPH: Augmentation Pressure/Pulse Height; AP: Augmented Pressure; AUC: area under the curve; DBP: diastolic blood pressure; HR: heart rate; LDI: laser doppler iontophoresis; MP: mean pressure; ms: milliseconds; PP: pulse pressure; PU: perfusion units; PWA: pulse wave analysis; SBP: systolic blood pressure; SNP: sodium nitroprusside.

Supplemental Table 8. Model of serum total cholesterol (TC) on women as a function of the logarithm of Glycoursodeoxycholic acid (GUDCA) and the Lithocholic acid (LCA). The model includes also the main study design factors: age, treatment, sequence, and period. The predictors have been centered and scaled.

Parameters	Estimate	df	t value	p-value (P(>|t|))
Intercept	6.045 ± 0.128	18.142	47.150	< 2e-16
Treatment effect (whole apple)	-0.313 ± 0.074	16.251	-4.221	0.001
Sequence Effect (control beverage)	-0.435 ± 0.258	18.387	-1.689	0.108
Period Effect (P1)	0.067 ± 0.075	16.262	0.897	0.383
Age (centered, scaled)	0.537 ± 0.292	19.334	1.837	0.082
GUDCA (centered, scaled)	-0195 ± 0.136	23.322	-1.430	0.166
LCA (centered, scaled)	0.621 ± 0.170	28.081	3.646	0.001
Interaction between Treatment effect (whole apple) and Age (centered, scaled)	-0.509 ± 0.166	16.191	-3.077	0.007
Interaction between Treatment effect (whole apple) and GUDCA (centered, scaled)	-0.457 ± 0.173	17.213	-2.644	0.017

Mean value ± SE. Significantly different when P<0.05

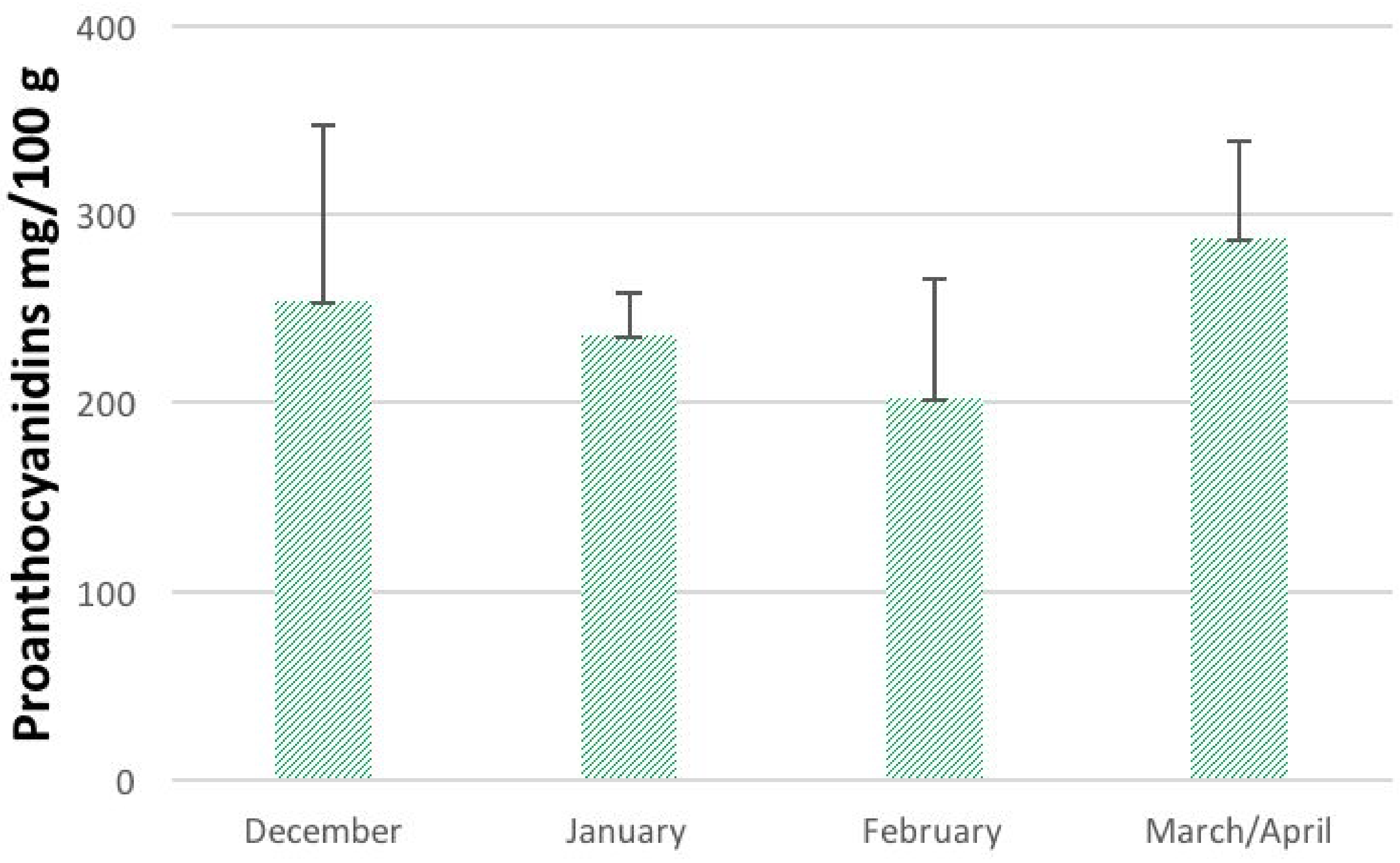

Supplemental Figure 1. Composition of oligomeric proanthocyanidins (PAs) (mg/100g) throughout the intervention period. Values are means ± SD.

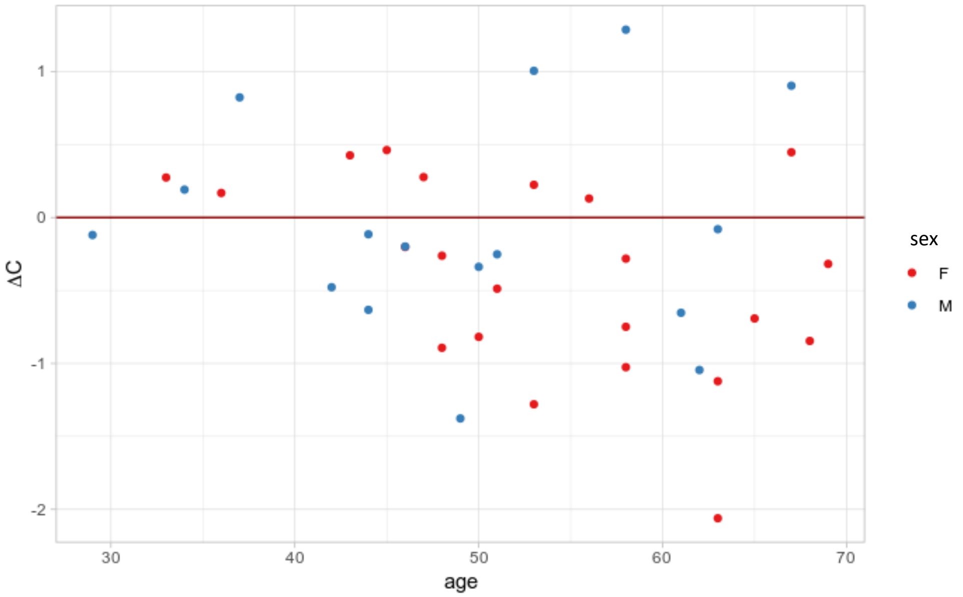

Supplemental Figure 2. Simplified model to explore the dependence of total cholesterol for every individual, represented as ΔC mmol/l= (C_{apple} – $C_{apple\ baseline}$) – ($C_{control}$ – $C_{control\ baseline}$), on age. Sequence and period effects were disregarded; no carryover was assumed. Age is shown in years. Every dot represents one volunteer (n=38 volunteers).

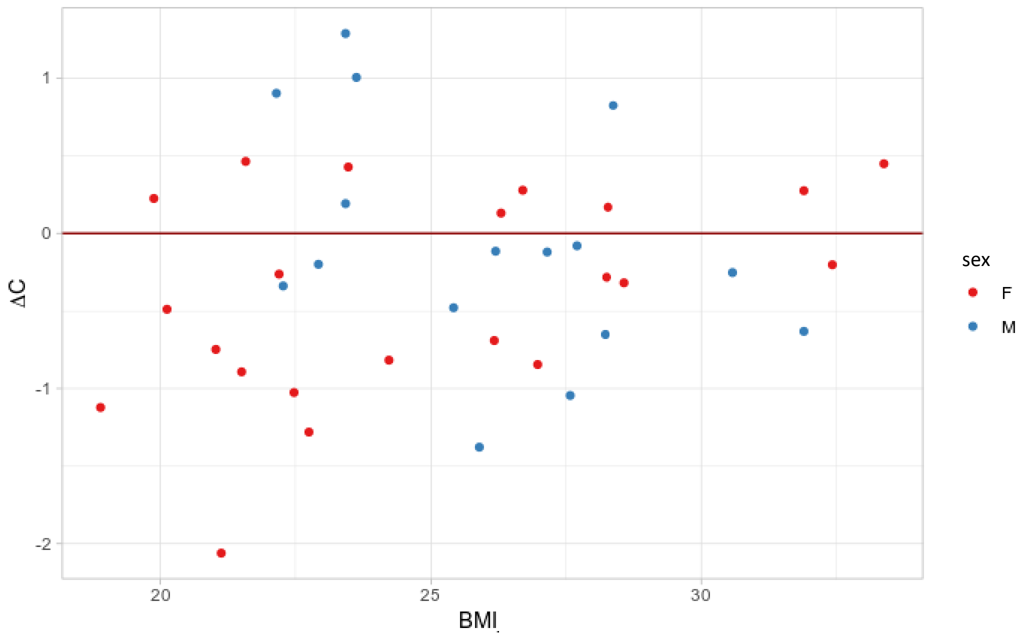

Supplemental Figure 3. Simplified model to explore the dependence of total cholesterol for every individual, represented as ΔC mmol/l= (C_{apple} – $C_{apple\ baseline}$) – ($C_{control}$ – $C_{control\ baseline}$), on BMI. Sequence and period effects were disregarded; no carryover was assumed. BMI is shown in kg/m^2. Every dot represents one volunteer (n=38 volunteers).

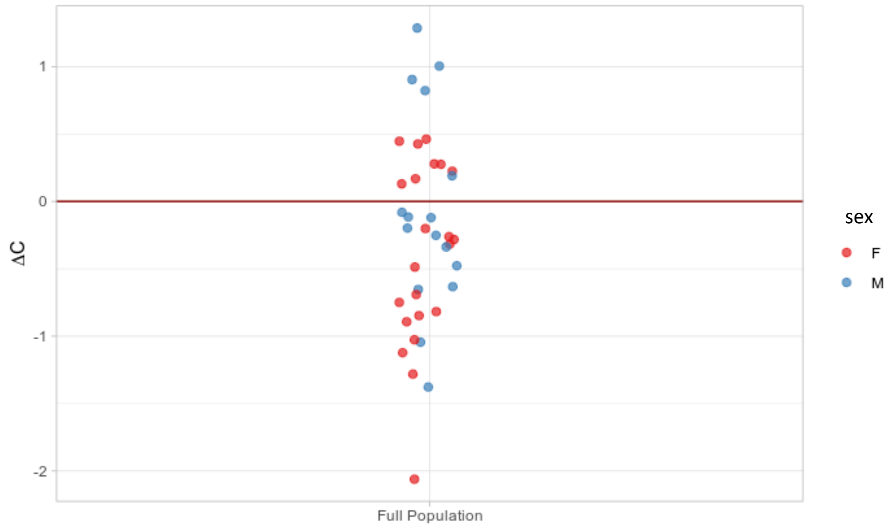

Supplemental Figure 4. Simplified model to explore the dependence of total cholesterol for every individual, represented as ΔC mmol/l= (C_{apple} – $C_{apple\ baseline}$) – ($C_{control}$ – $C_{control\ baseline}$), on sex. Sequence and period effects were disregarded; no carryover was assumed. Every dot represents one volunteer (n=38 volunteers). One sample t-test, with a null hypothesis of ΔC = 0, showed that whole apple consumption significantly reduced total cholesterol compared to the control beverage (P=0.03) on the full data set (n=38 volunteers). Visual inspection suggests that the level of response to the intervention could be different for men (n=16) and women (n=22). One sample t-test (H0, ΔC = 0), supports this observation (p-value = 0.723 for males, p-value = 0.012 for females).

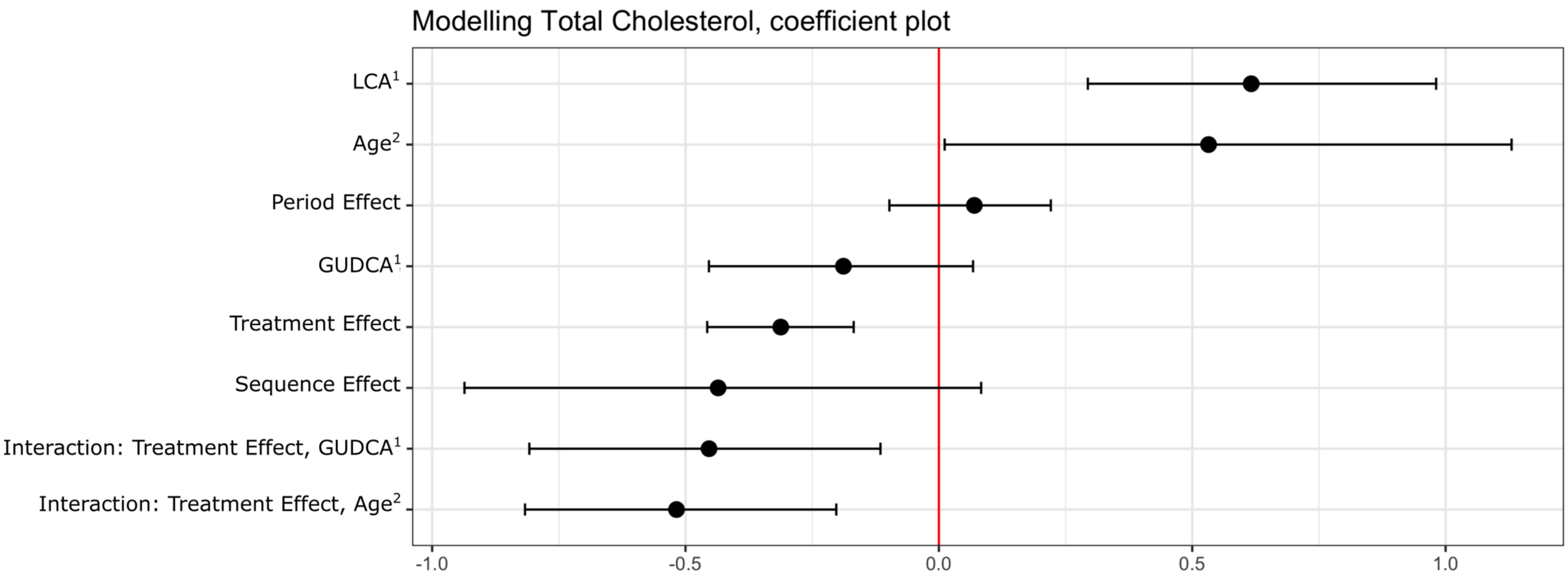

Supplemental Figure 5. Coefficient plot of the mixed linear model of TC on female: mean values (dots) and bootstrap confidence intervals (horizontal black lines) are reported. The vertical red line corresponds to 0. Confidence intervals not crossing the red line are considered statistically significant. [1] log10-transformed, scaled and centered variable; [2] scaled and centered variable.
